# High prevalence and plasmidome diversity of *optrA*-positive enterococci in a Shenzhen community, China

**DOI:** 10.3389/fmicb.2024.1505107

**Published:** 2024-12-20

**Authors:** Yulin Fu, Zhaoju Deng, Yingbo Shen, Weizhou Wei, Qiumei Xiang, Zhiyang Liu, Kunning Hanf, Suli Huang, Zexun Lv, Tingting Cao, Changfeng Peng, Rong Zhang, Xuan Zou, Jianzhong Shen, Stefan Schwarz, Yang Wang, Dejun Liu, Ziquan Lv, Yuebin Ke

**Affiliations:** ^1^Shenzhen Centre for Disease Control and Prevention, Shenzhen, China; ^2^National Key Laboratory of Veterinary Public Health Safety, College of Veterinary Medicine, China Agricultural University, Beijing, China; ^3^Shenzhen Hospital of Guangzhou University of Chinese Medicine (Futian), Shenzhen, China; ^4^Siming Centre for Disease Control and Prevention, Xiamen, China; ^5^Beijing University of Chinese Medicine Shenzhen Hospital (Longgang), Shenzhen, China; ^6^Department of Neurology, Shenzhen People's Hospital, Shenzhen, China; ^7^School of Public Health, Shenzhen University Medical School, Shenzhen University, Shenzhen, China; ^8^Department of Clinical Laboratory, the Second Affiliated Hospital, Zhejiang University School of Medicine, Hangzhou, China; ^9^Institute of Microbiology and Epizootics, Center for Infection Medicine, School of Veterinary Medicine, Freie Universität Berlin, Berlin, Germany; ^10^Veterinary Centre for Resistance Research (TZR), School of Veterinary Medicine, Freie Universität Berlin, Berlin, Germany

**Keywords:** oxazolidinone, antibiotic resistance, *optrA*, *Enterococcus*, plasmid, pAD1 + DOp1, *rep*9, *rep*US1

## Abstract

**Background:**

The emergence of *optrA*, which can confer resistance to phenicols and oxazolidinones in *Enterococcus* spp., poses a growing public health threat.

**Methods:**

102 *optrA*-positive enterococci (OPEs) including various species were isolated from feces of 719 healthy volunteers in a Shenzhen community, China. Antimicrobial susceptibility of these isolates was tested. Whole-genome sequencing and bioinformatics analysis were performed to characterize molecular epidemiology of OPEs.

**Results:**

Compared to *optrA*-negative enterococci (ONEs), antimicrobial resistance (linezolid, florfenicol, doxycycline, erythromycin and ciprofloxacin) and presence of antimicrobial resistance genes (ARGs) (*fexA*, *cat*, *tet*(M), *erm*(A), *erm*(B) and etc) were higher in OPEs. Phylogenetic analysis revealed that high similarly (19–338 SNPs) was observed between the *optrA*-positive *E. faecalis* from community and the strains from patients, animals, and environment. In 102 OPEs, the *optrA* gene was detected on the chromosome (*n* = 36), on plasmids (*n* = 62), or both (*n* = 4). A diverse range of *optrA*-carrying plasmid types was identified. The *rep*9-plasmid replicons were widely detected in *E. faecalis* (44/66), whereas *rep*US1-plasmid replicons were widely identified in other enterococcal species (7/66). Most of all ARGs harbored by isolates were co-existed on *optrA*-carrying plasmids, suggesting that the acquisition of *optrA*-carrying plasmids will pose a greater threat to public health. Notably, the pAD1 (*rep*9 family) + DOp1-type plasmids should receive more attention for the transfer of *optrA* given their high prevalence (36.36%), high number of co-located ARGs with *optrA* (83.87% of total ARGs) and presence in multiple sources. Tn*6674*, IS*1216E*, IS*Enfa1* and IS*Enfa5* are related to the transfer of chromosomal and plasmids-derived *optrA*, respectively. The *bcrABDR* gene cluster, *fexA*, and *erm*(A) were frequently identified surrounding *optrA* and may be transferred with *optrA* via IS*1216E* or IS*Enfa1*.

**Conclusion:**

The transfer of *optrA* gene is related to a variety of mobile elements (including plasmids, insertion sequences, transposons), which will promote the horizontal transfer of *optrA*. Moreover, many ARGs co-exist with *optrA* and could co-transfer with *optrA*. The acquisition of OPEs and *optrA*-carrying plasmids will pose a greater threat to public health and should be obtained more attention, especially *optrA*-positive *E. faecalis* and pAD1 + DOp1-type plasmids.

## 1 Introduction

Enterococci are Gram-positive, resilient bacteria that are widely distributed in the natural environment and commonly found in the intestinal tracts of animals; they can be easily isolated from a wide range of hosts (Fiore et al., 2019; Kang et al., 2019). Enterococci, particularly *Enterococcus faecalis* and *E. faecium*, are opportunistic pathogens that are among the main causes of human nosocomial infections, including urinary tract infections, intra-abdominal infections, surgical infections and even life-threatening conditions, such as endocarditis, septicemia, and bacteremia (Fiore et al., 2019; Sood et al., 2008). Moreover, *Enterococcus* spp. exhibit intrinsic resistances to several antimicrobials, such as cephalosporins, sulphonamides, clindamycin, and low concentrations of aminoglycosides. In addition, enterococci can acquire mobile genetic elements carrying antimicrobial resistant genes (ARGs), especially those against vancomycin, which limits the treatment options for infections caused by these bacteria (Zou et al., 2020; Xiong et al., 2022; Ben Braiek and Smaoui, 2019).

Linezolid, the first commercially available oxazolidinone, was approved by the Food and Drug Administration (FDA) in 2000. It is considered as a last-resort drug for treating severe infections caused by Gram-positive bacteria, such as vancomycin-resistant enterococci (VRE), methicillin-resistant staphylococci (MRSA) and multidrug-resistant pneumococci (Sadowy, 2018; Hashemian et al., 2018; Bain and Wittbrodt, 2001). Over the past decades, the prevalence of linezolid-resistant *E. faecalis* and *E. faecium* has increased in hospitals worldwide, posing a significant challenge to the treatment of VRE (Kang et al., 2019; Vorobieva et al., 2017). Transferable resistance to linezolid is based on the multi-resistance gene *cfr* (Schwarz et al., 2000), and the ATP-binding cassette (ABC)-F protein encoding genes *optrA* and *poxtA* (Wang et al., 2015; Antonelli et al., 2018). The multi-resistance gene *cfr* could encode a 23S rRNA methyltransferase that mediates resistance to five classes of antimicrobials (phenicols, lincosamides, oxazolidinones, pleuromutilins and streptogramin A) (Long et al., 2006). Moreover, several variants of *cfr* had been reported, including *cfr*(B), *cfr*(C), *cfr*(D) and *cfr*(E) (Guerin et al., 2020), of which only *cfr*(B) and *cfr*(D) could been detected in enterococci by searching NCBI pubmed. And unlike *poxtA*, which can cause bacteria to decrease susceptibility to phenicols, oxazolidinones and tetracyclines (Antonelli et al., 2018), *optrA* only confers resistance or decreased susceptibility to phenicols and oxazolidinones. However, *optrA* can also cause bacterial resistance to tedizolid, a second-generation oxazolidinone that was approved by the FDA in 2014 (Wang et al., 2015; Locke et al., 2014; Zhanel et al., 2015). Among the three transferable linezolid resistance genes, *optrA* has been the main driver of the recent increase in the prevalence of linezolid-resistant enterococci (LRE) in humans (Freitas et al., 2020). Since its first identification in enterococci from both humans and animals in 2015, *optrA* has been detected in various bacteria, including *Enterococcus*, *Staphylococcus*, *Streptococcus*, *Clostridium*, *Campylobacter*, *Fusobacterium*, *Listeria*, and *Salmonella* (Schwarz et al., 2021; Brenciani et al., 2022). Compared to the high prevalence of *cfr* in staphylococci, *Enterococcus* spp., such as *Enterococcus faecalis*, are the major host bacteria carrying *optrA* (Schwarz et al., 2021). According to NCBI Nucleotide databases, the gene *optrA* is present in bacteria (mainly *Enterococcus* spp.) from six continents, originating from humans, animals, animal-derived food, vegetables, and environmental sources (Schwarz et al., 2021). Reports have indicated that *optrA*-carrying LRE have been circulating globally in hospitals since at least 2005 (Deshpande et al., 2018).

While *optrA* has been distributed worldwide, especially in enterococci of animal origin, and *optrA*-positive enterococci (OPEs) pose an increasing threat to public health (Brenciani et al., 2022), the molecular characteristics of OPEs from humans have been rarely reported. Cai et al. screened for *optrA*-positive bacteria isolated from healthy individuals in Hangzhou, China in 2015 and 2022 (Cai et al., 2019; Shen et al., 2022; Shen et al., 2024), and Nüesch-Inderbinen et al. isolated enterococci harboring oxazolidinone resistance genes among healthy humans in a Swiss community in 2021 (Nuesch-Inderbinen et al., 2022). In our previous study, we found that 18.10% (102/565, 95% CI: 14.90–21.20%) of individual fecal samples were positive for *optrA* in a large community (>20,000 residents) in Shenzhen from 2018 to 2019 (Xiang et al., 2022), and suggested that higher daily consumption of pork (>50 g/day) [Odds ratio (OR) = 1.62, 95% CI: 1.02–2.57, *p* = 0.042] and hospitalization within 3 months (OR = 11.55, 95% CI: 2.15–61.96, *p* = 0.004) were associated with a higher risk of *optrA* carriage. On the basis of the previous study, we compared the antimicrobial resistance phenotypes and genotypes between OPEs and *optrA*-negative enterococci (ONEs) at present study. We also deciphered the *optrA*-carrying plasmids, the genetic environment of *optrA*, and phylogenetic relationships among *optrA*-positive enterococci using based on whole-genome sequencing (Illumina and GridION platform) and bioinformatics analysis.

## 2 Materials and methods

### 2.1 Sample collection and isolation of OPEs and ONEs

A total of 719 volunteers were recruited for sample collection from 1^st^ of March, 2018 to 31^st^ of December 2019 in a community in Shenzhen, Guangdong province, China. Ethical approval (R2018021) was granted by the ethics committee of the Shenzhen CDC on 19 January 2018. In total, 565 individual fecal samples accompanied by complete questionnaire information were selected for this study, and the sample selection criteria have been described in our previous studies (Xiang et al., 2022; Lv et al., 2022), in which 102 (102/565, 18.10, 95%CI: 14.90–21.20%) fecal samples were positive for *optrA* (Xiang et al., 2022) and 102 non-duplicate OPEs were isolated from these samples. The OPEs were identified using the following process: in brief, samples were cultured in LB broth (Luqiao, Beijing, China), then PCR was applied to detect the *optrA* gene in those isolates using the primers *optrA*-F: 5’-GCACCAGACCAATACGATACAA-3′, *optrA*-R: 5’-TCCTTCTTAACCTTCTCCTTCTCA-3′ (annealing temperature 52°C, amplicon size 794 bp) (Li, 2016). The *optrA*-positive broth was subsequently enriched in enterococcal culture-medium (SP3954, Luqiao, Beijing, China) containing 10 μg/mL florfenicol (ECM + FFC10). The enrichment was further inoculated onto the same plates (ECM + FFC10). Colonies on selective agar were confirmed for *optrA* and the species of enterococcal colonies positive for the *optrA* gene were identified by MALDI-ToF MS (MALDI Biotyper, Bruker, Germany). Moreover, ONEs were also isolated from *optrA*-positive fecal samples, following the same procedure, except that florfenicol was not added to the medium.

### 2.2 Antimicrobial susceptibility testing

AST of enterococci was conducted using the broth microdilution method. Eleven antimicrobial agents were included: linezolid (LNZ, 64–0.25 μg/mL), florfenicol (FFC, 128–0.5 μg/mL), amoxicillin-clavulanate (A/C, 128/64–0.5/0.25 μg/mL), ampicillin (AMP, 128–0.5 μg/mL), ciprofloxacin (CIP, 64–0.25 μg/mL), daptomycin (DAP, 128–0.5 μg/mL), erythromycin (ERY, 128–0.5 μg/mL), tigecycline (TGC, 64–0.25 μg/mL), doxycycline (DOX, 128–0.5 μg/mL), nitrofurantoin (FM, 256-8 μg/mL) and vancomycin (VAN, 128–0.5 μg/mL). The antimicrobial microtiter plates were customized by local merchants (IN. KING, Tianjin, China) according to our requirements for dilution ranges of 11 antimicrobial agents. Antimicrobial resistance was interpreted according to the clinical breakpoints in EUCAST (version v13.1) guidelines and the CLSI document M100 (33rd ed). *E. faecalis* ATCC 29212 was included as a quality control strain.

### 2.3 Whole genome sequencing and analysis

Enterococcal genomic DNA was extracted using the HiPure Bacterial DNA Kit (Guangzhou Magen Biotechnology Co., Ltd., China). Whole Genome Sequencing (WGS) with PE150 short-reads sequencing strategy (a minimum of 150-fold coverage) for each isolate was performed using the Illumina Novaseq 6,000 sequencing platform. Long-read sequencing of *optrA*-positive enterococci was conducted on the Oxford Nanopore Technologies (ONT) GridION platform. Long-read libraries of *optrA*-positive enterococci were prepared using the Ligation Sequencing Kit (SQK-LSK110) with EXP-NBD104 and EXP-NBD114. The constructed library was then subjected to ONT long-read sequencing in R9.4.1 flow cells on the ONT GridION platform according to the manufacturer’s protocol. Short-reads *de-novo* assembly or short- and long-reads hybrid *de-novo* assembly were performed using Unicycler v0.4.8 (Wick et al., 2017). The resulting contig sets (chromosomal or plasmid) were annotated with Bakta v1.8.2 (Schwengers et al., 2021) (accessed on 1 July, 2024). Multilocus Sequence Typing (MLST) was identified using mlst v2.19.0 (Seemann, 2020a) based on assembled contigs. ARGs, Virulence factors genes (VFGs) and plasmid types were identified using Abricate v1.0.1 (Seemann, 2020b) (accessed on 1 July, 2024). Single-nucleotide polymorphisms (SNPs) were identified using Snippy v4.6.0 (Seemann, 2020c), and SNP distances between isolates were calculated based on concatenated SNPs using snp-dists v0.8.2 (Seemann and Klötzl, 2021). The population structure of those isolates was delineated using Bayesian model-based population structures (BAPs) analysis via Fastbaps v1.0.8 (Tonkin-Hill et al., 2019). Maximum likelihood based phylogenetic trees were constructed using IQ-TREE v2.2.2.7 (Minh et al., 2020). Phylogenetic trees were then visualized using Interactive Tree of Life (iTOL) v6.7.6 (Letunic and Bork, 2024) with the corresponding features of each isolate. Genetic characteristics of *optrA*-positive isolates were compared with BLAST Ring Image Generator (BRIG) 0.95 and Easyfig 2.2.5 (Sullivan et al., 2011; Alikhan et al., 2011). All *optrA*-harboring enterococcal genomes available in the NCBI database were downloaded on 7 April, 2024 for further analysis. Moreover, some *optrA*-carrying plasmid sequences in NCBI database were downloaded for comparison of plasmid structures (accessed on 10 July, 2024). To ascertain whether there are any novel *optrA*-carrying plasmids, all of the circular *optrA*-carrying plasmids identified herein were deposited in NCBI BLASTn and compared with the NCBI nonredundant (nr) database (accessed on 10 July, 2024). When the reference sequence contained *optrA*, but the coverage was <60%, or the reference sequences did not contain *optrA*, or the coverage and identity were both >90%, but the plasmid type was different, the respective plasmids have been considered as novel *optrA*-carrying plasmids.

### 2.4 Statistical analyses

The statistical analyses were conducted in R v.4.3.1 (R Core Team, 2023). Differences between the numbers of distinct ARGs/VFGs per isolate in different groups were assessed using the Kruskal-Wallis test. Gene prevalence differences between groups were assessed with the *χ*^2^ test (*n* ≥ 40) or Fisher’s exact test (*n* < 40 or minimum expected frequency < 1).

## 3 Results

### 3.1 High antimicrobial resistance rates in OPEs, especially *Enterococcus faecalis*

We previously identified 102 *optrA*-positive fecal samples from the community population and 102 non-duplicate *optrA*-positive enterococci were selected for further analysis (Xiang et al., 2022). *Enterococcus faecalis* (*n* = 75/102, 73.5%) was the predominant species among the OPEs, followed by *E. faecium* (*n* = 7/102, 6.9%), *E. avium* (*n* = 6/102, 5.9%), *E. casseliflavus* (*n* = 6/102, 5.9%), *E. gallinarum* (*n* = 4/102, 3.9%) and *E. hirae* (*n* = 4/102, 3.9%). Concurrently, for species matching, *optrA*-negative *E. faecalis* were also isolated from the 75 *optrA*-positive fecal samples from which we isolated *optrA*-positive *E. faecalis* because of its high prevalence rate. A total of 26 *optrA*-negative *E. faecalis* (paired samples) were selected from 26 *optrA*-positive *E. faecalis* fecal samples to explore the differences between *optrA*-positive (*optrA*+_Match, *n* = 26) and *optrA*-negative (*optrA*-_Match, *n* = 26) isolates.

**Figure 1:**
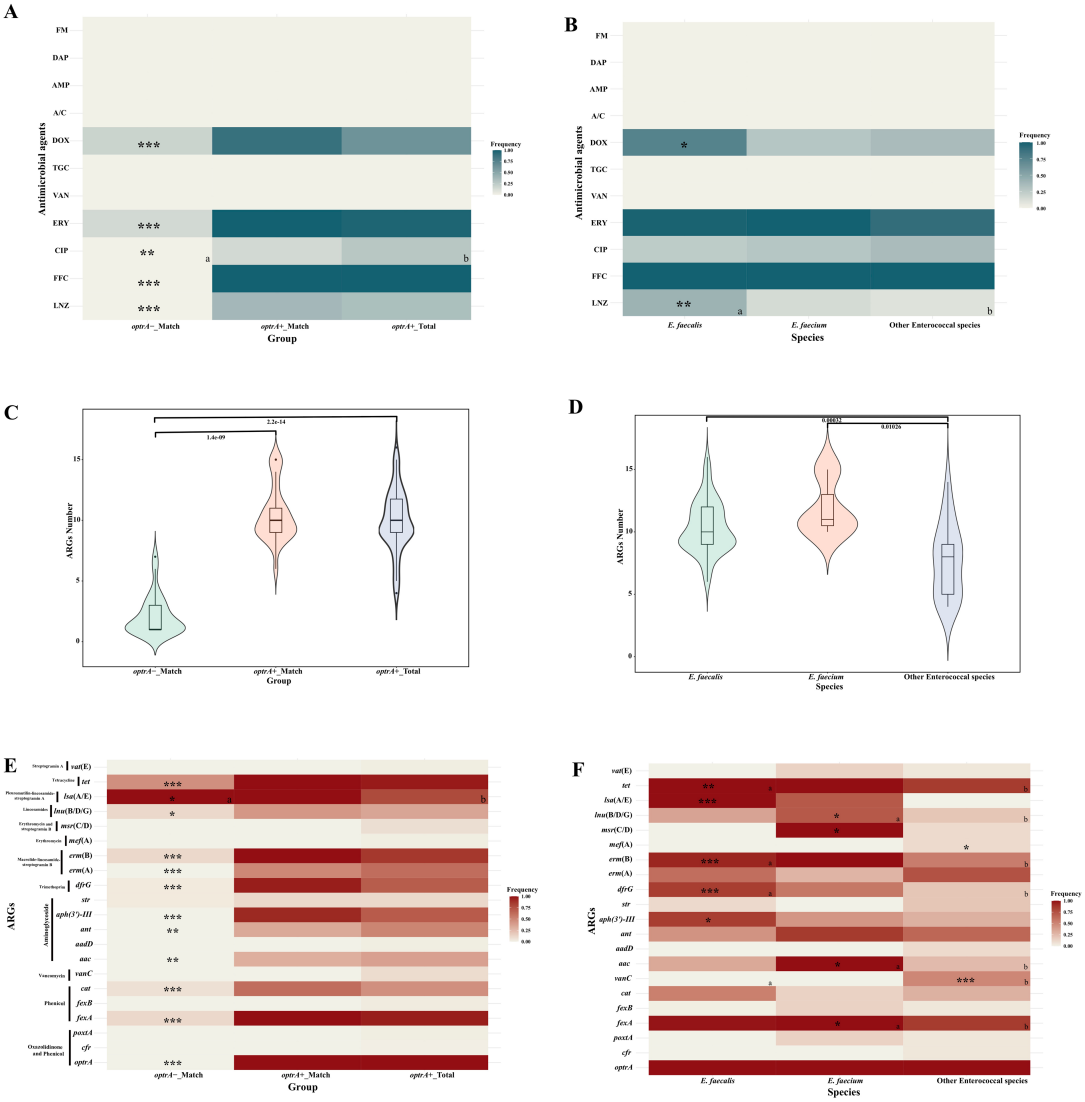
Comparison of antimicrobial susceptibility, ARGs numbers and profiles of OPEs and ONEs **(A,C,E)**, and of different enterococcal species **(B,D,F)**. *cat*--*cat* or *cat*(pC221); *vanC--vanC1XY*, *vanC2XY* or *van*C4XY; *aac--aac(6′)-Ii*, *aac(6′)-Iid*, or *aac(6′)-aph(2″)*; ant--*ant(6)-Ia* and *ant(9)-Ia*; *msr*(C/D)--*msr*(C) or *msr*(D); *lnu*(B/D/G)--*lnu*(B), *lnu*(D) or *lun*(G); *lsa*(A/E)--*lsa*(A) or *lsa*(E); *tet--tet*(L), *tet*(M), *tet*(O/W/32/O) or *tet*(S). **a and b** Represent significant differences between the two groups; only asterisks indicated that the group showed significant differences with other groups; “*”: *p* < 0.05; “**”: *p* < 0.01; “***”: *p* < 0.001.

All of the *optrA*-positive enterococci were resistant to florfenicol (102/102, 100%) and susceptible to vancomycin, tigecycline, amoxicillin-clavulanate, ampicillin, daptomycin and nitrofurantoin (Supplementary Tables S1, S2). The overall resistance rate for linezolid was 33.3% (34/102). Compared to *optrA*-_Match isolates, high resistance rates were observed in OPEs, with higher resistance rates against linezolid, florfenicol, doxycycline, erythromycin, and ciprofloxacin, in *optrA*+_Match (LNZ: 38.5%, FFC: 100%, DOX: 88.5%, ERY: 100%, CIP: 15.4%, *p* < 0.001, except for CIP) and all of OPEs in this study (*optrA*+_Total, *n* = 102) (LNZ: 33.3%, FFC: 100%, DOX: 63.7%, ERY: 97.0%, CIP: 27.5%, *p* < 0.001 for all but *p* < 0.01 for CIP; Figure 1A; Supplementary Table S2). We observed that the linezolid resistance rate varied in different enterococcal species, 41.3% (31/75) for *E. faecalis*, 14.3% (1/7) for *E. faecium* and 10.0% (2/20) for other enterococcal species (*E. avium*, *E. casseliflavus*, *E. gallinarum*, and *E. hirae*, Figure 1B; Supplementary Table S3). The resistance rates of different OPEs species to ciprofloxacin were relatively low (25.3–35.0%), but the erythromycin resistance rates were high (90.0–100%). Particularly, the doxycycline resistance rate was significantly higher in *E. faecalis* (56/75, 74.7%; *p* < 0.001) than in other enterococcal species.

### 3.2 High presence of ARGs in OPEs, especially *Enterococcus faecalis* and *Enterococcus faecium*

A total of 21 ARGs were identified in the different enterococcal species (Figures 1C,F; Supplementary Table S4). The number of ARG in *optrA*+_Match (median 10, interquartile range (IQR) 9–11) and *optrA*+_Total (median 10, IQR 9–11.75) isolates were distinctly higher than that in *optrA*-_Match isolates (median 1, IQR 1–3; *p* < 0.0001, Figure 1C). Notably, the phenicol resistance gene *fexA*, the aminoglycoside resistance genes *aac*, *ant* and *aph(3′)-III*, the trimethoprim resistance gene *dfrG*, the macrolide-lincosamide-streptogramin B (MLS_B_) resistance genes *erm*(A) and *erm*(B), the lincosamides resistance genes *lnu*(B/D/G), and the tetracycline resistance genes *tet* were more prevalent in *optrA*+_Match and *optrA*+_Total than that in *optrA*-_Match isolates, especially *fexA*, *cat*, *ant(9)-Ia*, *aph(3′)-III*, *erm*(A), *erm*(B), *lsa*(A), *tet*(L) and *tet*(M) (Figure 1E). Conversely, no significant differences in the 42 VFGs were observed between *optrA*+_Match or *optrA*+_Total and *optrA*-_Match isolates (Supplementary Figure S1A; Supplementary Table S5).

Among the different species, ARGs in *E. faecalis* (median 10, interquartile range (IQR) 9–12) and *E. faecium* (median 11, IQR 10.5–13) were more frequently observed than in other enterococcal species (median 8, IQR 5–9) (*p* < 0.001 and *p* < 0.05 respectively) (Figure 1D). Both *fexA* (85–100%) and *tet* (85–100%) were predominant in all of the *optrA*-positive species (Figure 1F). In addition, *poxtA* was found in one *E. faecium*, and *cfr* was identified in one *E. hirae* and one *E. avium* isolate. However, all three isolates carrying two types of linezolid resistance genes showed susceptibility to linezolid. Moreover, the prevalence of other ARGs in different *optrA*-positive enterococci varied substantially. The pleuromutilin-lincosamide-streptogramin A resistance gene *lsa*(A/E) was detected in all *E. faecalis* isolates, but less frequently in *E. faecium* (71.4%) and other enterococcal species (0%) (*p* < 0.001). The MLS_B_ resistance gene *erm*(B) was present in 93.3% *E. faecalis* and 100% *E. faecium* isolates, but at lower frequency (55%) in other enterococcal species. The vancomycin resistance gene clusters *vanC* were absent in either *E. faecalis* or *E. faecium*, but were found in 50% of other enterococcal species because *E. casseliflavus* and *E. gallinarum* are intrinsically resistant to vancomycin by carrying the *vanC* gene cluster on their chromosomes (Garcia-Solache and Rice, 2019) (*p* < 0.05). The aminoglycoside resistance gene *aac* and the erythromycin and streptogramin B resistance gene *msr*(C/D) were identified in all of the *E. faecium* isolates, but at a lower frequency in *E. faecalis* (25.0–34.6%) and other enterococcal species (0–10%). In addition, VFGs were more frequently detected in *E. faecalis* (median 25, IQR 17–26) than in *E. faecium* (median 1, IQR 0–1) and other enterococcal species (median 0, IQR 0–0) (*p* < 0.001, Supplementary Figures S1B,D).

### 3.3 Heterogeneous population structure of OPEs and ONEs

**Figure 2:**
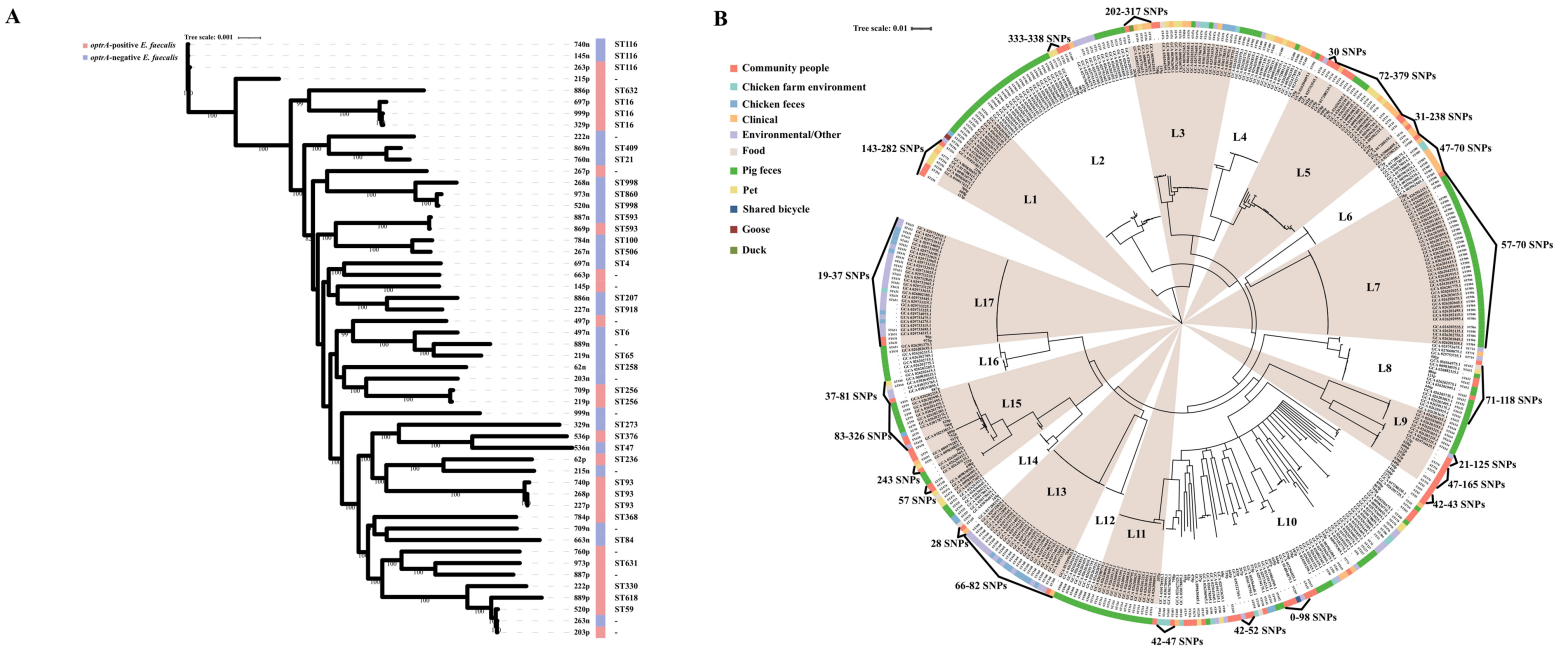
Phylogenetic trees based on maximum likelihood analysis of 26 pairs *optrA*-positive and -negative *E. faecalis* isolated from the same fecal samples **(A)**, and 414 *optrA*-positive *E. faecalis* including 75 strains isolated from the present study and 339 strains whose genomes were downloaded from NCBI **(B)**.

MLST revealed that both *optrA*-positive and *optrA*-negative *E. faecalis* isolates exhibited a heterogeneous population structure (Supplementary Table S6). Among the 75 *optrA*-positive *E. faecalis*, 52 isolates were assigned to 26 STs, including ST16 (*n* = 10, 13.3%), ST376 (*n* = 5, 6.6%), ST93 (*n* = 4, 5.3%), ST632/ST256 (*n* = 3, 4.0%), ST59/ST202/ST330/ST618/ST631/ST633 (*n* = 2, 2.6%), and 15 STs were represented by one isolate each, while the remaining 23 *optrA*-positive *E. faecalis* isolates belonged to novel STs. The STs differed across the *optrA*-positive and *optrA*-negative *E. faecalis* in 26 paired samples (Figure 2A). In addition, the *optrA*-positive and *optrA*-negative *E. faecalis* isolates were phylogenetically distant from each other (Figure 2A). Among seven *optrA*-positive *E. faecium*, ST66, ST55, ST944 and ST194 were identified in four isolates and novel STs in other three isolates. To explore the phylogenetic relationship between OPEs isolated from the tested community and other sources, we downloaded 945 whole genome sequences using the keywords “*optrA*” from NCBI and screened *E. faecalis* isolates from China with their sources clearly documented in the respective metadata. A total of 339 *E. faecalis* were included in the phylogenetic analysis along with the 75 *E. faecalis* isolates from this study (Figure 2B). The BAPs analysis divided these 414 *optrA*-positive *E. faecalis* isolates into 17 clusters with no prominent BAPs, suggesting a heterogeneous population structure of *optrA*-positive *E. faecalis*. In line with the phylogenetic analysis, high diversity was also found in MLST typing, 51.93% (215/414) of the isolates, belonging to nine STs, were obtained from different origins and only ST506 was predominately isolated from pigs (38/39). There was no significant correlation between neither the BAPs clusters nor the sequence types with the source of *optrA*-positive *E. faecalis*. However, high similarly between *optrA*-positive *E. faecalis* isolated from the study community and other origins (human clinics, pigs, chickens, pets, and environment) was observed in the same BAPs cluster according to low core genome diversity reflected by a limited number of SNPs (19–338 SNPs difference).

### 3.4 Location of *optrA* gene and diversity of *optrA*-carrying plasmid types

**Figure 3:**
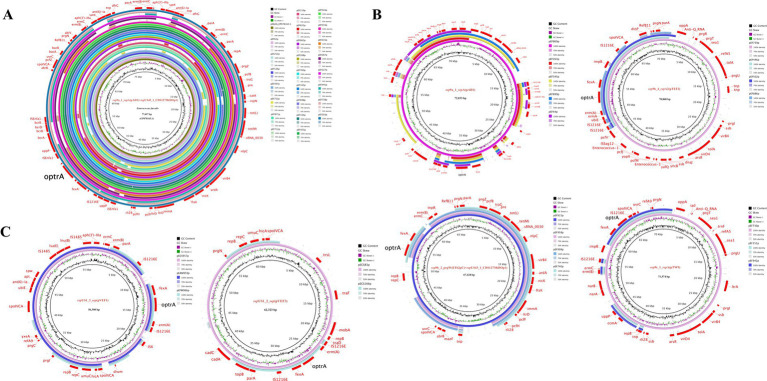
Circular comparison of *optrA*-carrying plasmids of the same type in this study. **(A)** pAD1 + DOp1; **(B)** *optrA*-carrying plasmids in *E. faecalis*; **(C)** *optrA*-carrying plasmids in other enterococcal species.

To investigate the genetic basis of *optrA* dissemination among different isolates, all of the 102 OPEs underwent sequencing via a combination of short-read and long-read WGS, followed by hybrid assembly. The majority of hybrid assemblies produced complete *optrA*-carrying sequences and only 11 *optrA*-carrying sequences were incomplete or non-circular (Supplementary Table S6). The *optrA* was detected on on the chromosome (*n* = 36), on plasmids (*n* = 62), or both (*n* = 4, three *E. faecalis* and one *E. gallinarum*, Table 1). A total of 15 distinct plasmid types were identified among 66 *optrA*-carrying plasmids with sizes varying between 25.9 and 92.8 kb. This highlighted the considerable diversity of *optrA*-carrying plasmids among enterococci isolated from the community population (Table 1; Supplementary Table S6). The types of replicons carried by different enterococci varied considerably. Half of the *optrA*-harboring plasmids (33/66) carried multiple replicons, predominant with *rep*9a_1_*rep*A(pAD1) + *rep*US43_1_CDS12738(DOp1) (24/66, 36.36%), which were identified exclusively in *E. faecalis*. One plasmid harbored even three replicons, *rep*33_1_*rep*(pSMA198) + pAD1 + DOp1. The *rep9* (belonging to *rep*A_N) was the mostly detected replication gene (44/66), followed by *rep*US43 (belonging to *rep*_trans; 29/66), both of which were only identified in *E. faecalis*. In addition, *rep*US1, associated with the broad host-range plasmids Inc18, was identified in seven *optrA*-carrying plasmids derived from three *E. casseliflavus*, two *E. avium*, one *E. gallinarum* and one *E. faecium* isolate. The backbones of 19 out of 24 pAD1 + DOp1 *optrA*-carrying plasmids all showed high homology (coverage 100% and identity >99.90%) with plasmid pNS2A, located in a Chinese water-borne *E. faecalis* isolate (GenBank no. CP078163.1, Figure 3A). Most of the pAD1 + DOp1 plasmids carried additional ARGs including *ant(6)-Ia*, *aph(3′)-III*, *cat*, *fexA*, *dfrG*, *erm*(B), *tet*(L), *tet*(M) and etc. And the bacitracin resistance cluster *bcrABDR* was also frequently identified on this type *optrA*-carrying plasmids through annotation of plasmid sequences and NCBI BLASTn. The backbones of other type plasmids with identical replicons were dissimilar, and only *rep*9b_2_prgW(EF62pC) + DOp1-type and *rep*US1_3_*rep*(pVEF1)-type *optrA*-carrying plasmids showed relatively high homology (Figures 3B,C). Furthermore, putative *rep* genes were identified on seven circular-formed *optrA*-carrying segments but no clear replicons were assigned, which would necessitate further investigation.

**Table 1:** Location of the *optrA* gene as well as typing and sizes of *optrA*-carrying plasmids.

*optrA* Location (Number)^a^	Species (Number)	Plasmid typing (Number)	Plasmid size	Circular
C + p(4)	*E. faecalis*(3)	*rep*9a_1_*rep*A(pAD1) + *rep*US43_1_CDS12738(DOp1) (1)	length = 58.6 kb	Yes
		*rep*US26_1_EFD32pB0001(EFD32pB) (1)	length = 52.9 kb	Yes
		None(1)	length = 8.2 kb	No
	*E. gallinarum*(1)	*rep*US1_1_*rep*E(DOp2) (1)	length = 59.8 kb	Yes
C(36)	*E. faecalis*(19)	–	–	Yes(18) + No(1)
	*E. faecium*(6)	–	–	Yes
	*E. avium*(4)	–	–	Yes(3) + No(1)
	*E. gallinarum*(3)	–	–	Yes(2) + No(1)
	*E. hirae*(4)	–	–	Yes
p(62)	*E. faecalis*(53)	*rep*9a_1_*rep*A(pAD1) + *rep*US43_1_CDS12738(DOp1) (23)	length = 40.6–87.8 kb	Yes(22) + No(1)
		*rep*9a_1_*rep*A(pAD1) (5)	length = 38.6–72.0 kb	Yes
		*rep*9b_2_prgW(EF62pC) + *rep*US43_1_CDS12738(DOp1) (4)	length = 42.6–67.1 kb	Yes(3) + No(1)
		*rep*9b_4_*rep*A2(pTEF2) (4)	length = 25.9–70.9 kb	Yes
		*rep*9c_1_*rep*A(pTW9) (3)	length = 35.9–71.6 kb	Yes
		*rep*33_1_*rep*(pSMA198) + *rep*9a_1_*rep*A(pAD1) + *rep*US43_1_CDS12738(DOp1) (1)	length = 81.8 kb	Yes
		*rep*9a_1_*rep*A(pAD1) + *rep*27_2_*rep*A(pSGG1) (1)	length = 74.0 kb	Yes
		*rep*9b_2_prgW(EF62pC) (1)	length = 82.0 kb	Yes
		*rep*9b_2_prgW(EF62pC) + *rep*33_1_*rep*(pSMA198) (1)	length = 92.8 kb	Yes
		None(10)	length = 6.6–87.9 kb	Yes(6) + No(4)
	*E. faecium*(1)	*rep*US1_3_*rep*(pVEF1) (1)	length = 56.7 kb	Yes
	*E. avium*(2)	*rep*1_6_*rep*E(pTEF1) + *rep*US1_3_*rep*(pVEF1) (1)	length = 64.5 kb	Yes
		*rep*US1_3_*rep*(pVEF1) (1)	length = 51.4 kb	Yes
	*E. casseliflavus*(6)	*rep*US1_2_*rep*(pVEF3) (2)	length = 30.3–62.3 kb	Yes
		*rep*US1_3_*rep*(pVEF1) (1)	length = 56.3 kb	Yes
		*rep*US28_*rep*E(pQY182) (1)	length = 53.8 kb	Yes
		None(2)	length = 24.5–60.2 kb	Yes(1) + No(1)

^aC, Chromosome; P, Plasmid.^

### 3.5 Novel *optrA*-carrying plasmids were identified and most ARGs were co-located on *optrA*-carrying plasmids

To investigate *optrA*-carrying plasmids, all of the circular *optrA*-carrying plasmids identified in this study were deposited in NCBI BLASTn and compared with the NCBI nr database (accessed on 10 July, 2024). The pAD1 + DOp1 *optrA*-carrying plasmids were not only prevalent in the community population but also showed highly homology (coverage >90% and identity >99%) with plasmids from various sources (water, patient urine, cattle, feces, pig, pet food, wastewater and other environments) deposited in NCBI (GenBank accession no. CP078163.1, CP145115.1, CP116554.1, CP116558.1, CP088199.1, CP097007.1, CP116556.1, CP096048.1, MK784777.1, MK465704.1, CP148053.1, CP118757.1, OP046178.1, etc.). Moreover, these plasmids were predominantly found in *E. faecalis*, with only MK465704.1 identified in *E. faecium*. Notably, we identified 16 novel *optrA*-carrying plasmids with their sizes ranging from 30 kb to 92 kb and various replicon types or combinations of replicons. Moreover, circular comparisons of 16 novel *optrA*-carrying plasmid maps were conducted with the most similar plasmid sequences in the NCBI nr database (Supplementary Figures S2–S4). Among the novel plasmids, the *optrA*-carrying plasmid pEF575p harboring three replicons (pSMA198 + pAD1 + and DOp1) was detected in *E. faecalis*. We found that the plasmid pEF575p also showed high similarly with plasmid pNS2A (coverage 89% and identity 99.97%) which same as pAD1 + DOp1-type *optrA*-carrying plasmids (Supplementary Figure S2A). And in this study, nine circular *optrA*-carrying plasmids were identified in non-*E. faecalis* enterococcal species. The result of BLASTn with NCBI nr database revealed that seven were novel, with six carrying the *rep*US1 replicon (Supplementary Figure S3).

**Figure 4:**
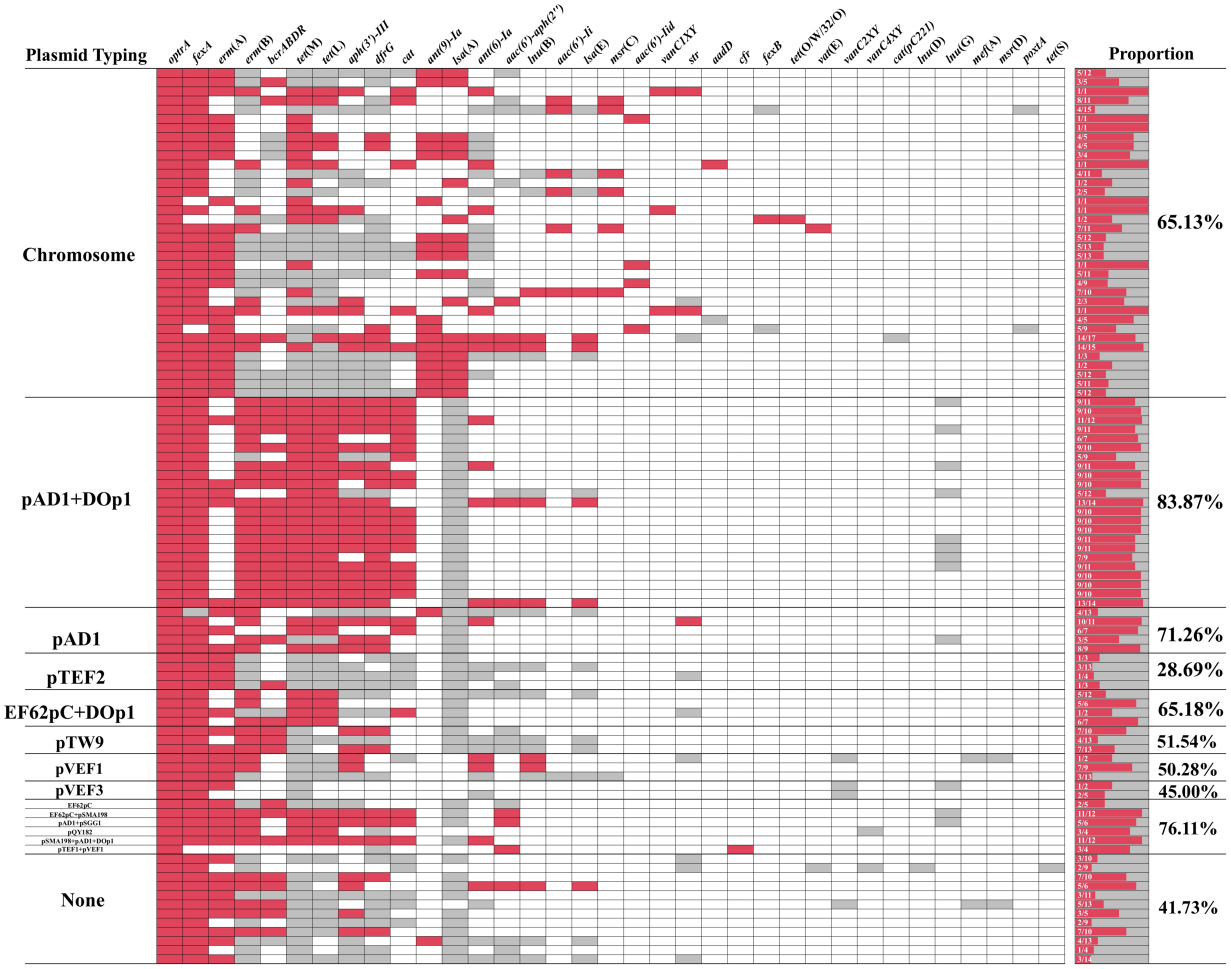
The heatmap of ARGs coexisting with *optrA*. Red indicates that the ARGs and *optrA* co-exist on the same contig, gray indicates that the ARGs and *optrA* do not co-exist on the same contig, and white indicates that the ARGs were not detected in the same isolate.

**Figure 5:**
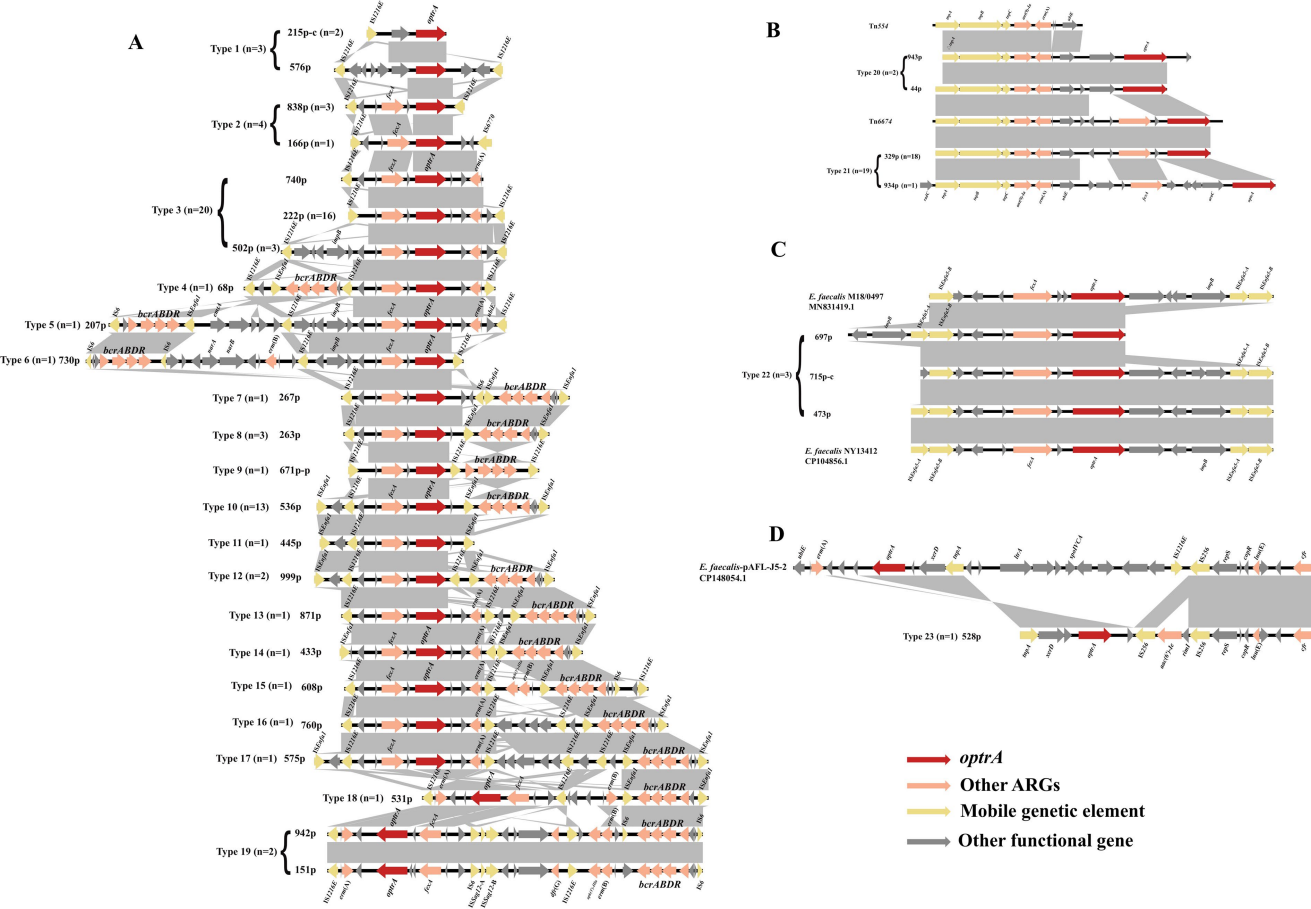
Schematic presentation and comparison of the genetic environment of *optrA* in the enterococci investigated in this study. The “-c” in the number indicates that the sequence is derived from the chromosome sequences, and other sequences are derived from the plasmid sequences. **(A)** IS*1216E*-related; **(B)** Tn*554*-related; **(C)** IS*Enfa5*-related; **(D)** *optrA* and *cfr* co-existing in the same genetic environment.

Multiple ARGs were identified on these *optrA*-carrying plasmids and the heatmap showed that 28.69–83.87% of ARGs were co-located with *optrA*-carrying plasmids, except for four isolates with *optrA* located both on the chromosome and plasmid in one isolate (Figure 4). The ARGs co-located with *optrA* on pAD1 + DOp1, pAD1, EF62pc + DOp1, pTW9, pVEF1 and other types of *optrA*-carrying plasmids (including EF62pC, EF62pC + pSMA198, pAD1 + *rep*27_2_*rep*A(pSGG1), *rep*US28_*rep*E(pQY182), pSMA198 + pAD1 + DOp1, and *rep*1_6_*rep*E(pTEF1) + pVEF1), accounted for more than 50% of the total ARGs carried by the whole genomes of the isolates. The genes *fexA* (60/62, 96.77%) was the ARGs most commonly co-located with *optrA*-carrying plasmids, followed by *erm*(B) (42/62, 67.74%), *bcrABDR* (34/62, 54.84%), *tet*(M) (34/62, 54.84%), *tet*(L) (34/62, 54.84%), *erm*(A) (33/62, 53.23%), *aph(3′)-III* (32/62, 51.61%), *dfrG* (30/62, 48.39%), *cat* (24/62, 38.71%) and other ARGs. The results demonstrated that the acquisition of *optrA*-carrying plasmids facilitates host strains to harbor more ARGs, which poses a significant threat to human health. Notably, up to 83.87% of ARGs were found to be co-located in pAD1 + DOp1-type plasmids, emphasizing the need for increased attention to this type *optrA*-carrying plasmids. Furthermore, the co-existence of *optrA* and *cfr* genes was observed in a novel plasmid pEAM528p from one *E. avium* isolate and two replicons pTEF1 and pVEF1, both belonging to the broad host Inc18 plasmids, were identified in pEAM528p. This plasmid exhibited a high degree of homology (coverage 75% and identity 99.30%) to the pTEF1-type plasmid pAFL-J5-2, which was carried by porcine *E. faecalis* isolated from Xinjiang, China (GenBank accession number: CP148054.1, Supplementary Figure S4F). Especially, the flanking region of *optrA* in pEAM528p was highly similarly to that in pAFL-J5-2, and the only difference was the presence of an aminoglycoside resistance gene *aac(6′)-Ie* in pEAM528p (Figure 5D).

### 3.6 *optrA* is commonly surrounded by mobile genetic elements

The genetic contexts of *optrA* (*n* = 106) identified in this study can be categorized into four classes and 27 types: IS*12126E*-related (types 1–19, *n* = 59, Figure 5A), Tn*554*-related (types 20–21, *n* = 21, Figure 5B), IS*Enfa5*-related (type 22, *n* = 3, Figure 5C), *cfr* located in the surrounding of *optrA* (type 23, *n* = 1, Figure 5D) and no MGEs (type 24: *fexA*-*optrA*, *n* = 12; type 25: *optrA*-*erm*(A), *n* = 1; type 26: *fexA-optrA-erm*(A), *n* = 8; type 27: *optrA*, *n* = 1). The IS*12126E*-related context of *optrA* was the most prevalent (59/106, 55.66%). IS*1216E* can be located upstream or downstream of *optrA*, or both in the same or different orientations. Besides IS*1216E*, the IS*Enfa1* often appears in the surrounding area of *optrA* as IS*Enfa1*-*bcrABDR*-IS*Enfa1* (Figure 5A, types 7, 8, 10, 12–14, 16–18; 22/59). Both IS*Enfa1* and IS*1216E* belong to the IS*6* family, with IS*Enfa1* exhibiting 94% amino acid identity to IS*1216E*. The bacitracin resistance cluster *bcrABDR* has been identified in *E. faecalis* previously and frequently detected in surrounding genetic context of *optrA* in present study (Matos et al., 2009). It can also appear as IS*1216E*-*bcrABDR*-IS*1216E* structure (type 9). Moreover, IS*1216E* and IS*Enfa1* were interspersed between *optrA* and *bcrABDR* in various forms (types 4–19). Besides the IS*1216E*, Tn*554* or the Tn*554*-related transposon Tn*6674* were commonly associated with *optrA* (Figure 5B, type 21, *n* = 19). Tn*6674* can be active and form circular intermediates to accelerate the transfer of *optrA* (Li et al., 2019). In addition, IS*Enfa5* in the upstream region or both up- and downstream regions of *optrA* were observed in three *optrA*-carrying contigs (Figure 5C). Moreover, florfenicol resistance gene *fexA* and MLS_B_ resistance gene *erm*(A) were frequently detected in most of genetic contexts surrounding *optrA*.

**Figure 6:**
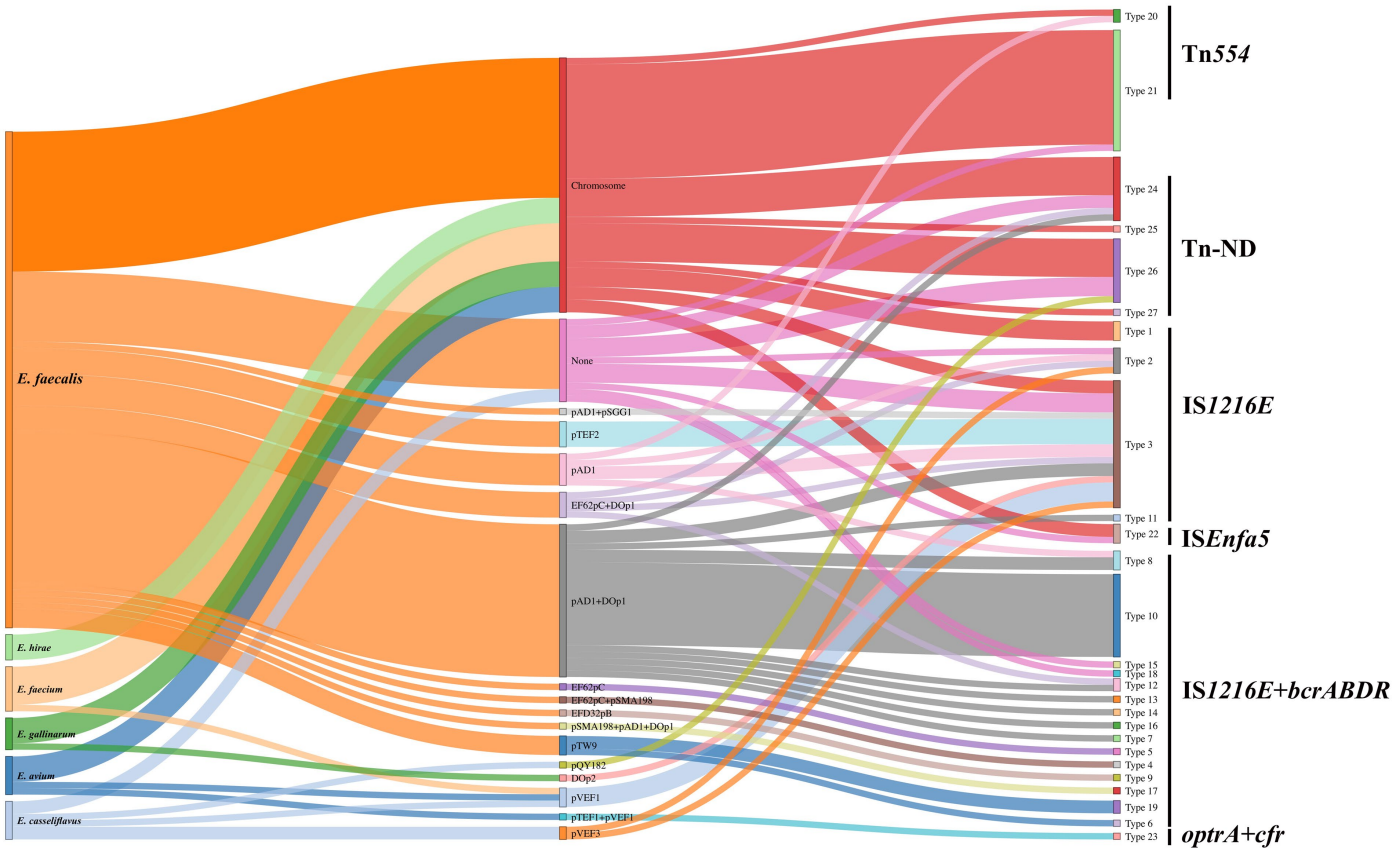
Sankey diagram displaying the flow of enterococcal species, plasmid types, and genetic environment of *optrA*. None indicated no known replicons were detected. Tn-ND indicated transposons were not identified in the genetic environment of *optrA*.

A sankey diagram was employed to analysis the relationships among enterococcal species, plasmid types, and genetic environments of *optrA* (Figure 6). The results showed that *optrA* was predominantly identified on plasmids (53/72, 73.61%) in *E. faecalis* and on chromosomes in other enterococci (17/26, 65.38%). Concurrently, most plasmid types (10/15) were identified in *E. faecalis*, in contrast to only five types were found in other enterococci. Tn*554*-related genetic contexts of *optrA* and Tn-ND (MGEs were absent in the surrounding area of *optrA*) were primarily located on chromosome. IS*1216E*-related genetic contexts of *optrA* could be located on the chromosome or plasmids, yet mostly on plasmids. IS*1216E* + *bcrABDR*-related genetic contexts of *optrA* could be located on a variety of plasmids and were mainly detected in *E. faecalis* (with occasional detection in *E. casseliflavus*).

## 4 Discussion

To date, most studies have focused on OPEs from animals or the environment, with few studies systematically exploring these isolates in healthy humans. The prevalence of *optrA* in a community population in Shenzhen (18.1%) (Xiang et al., 2022) was higher than that in a healthy population in Switzerland (3.8%) (Nuesch-Inderbinen et al., 2022), but similar to that in Hangzhou, China in 2022 (19.3%) (Shen et al., 2022). According to the China Antimicrobial Surveillance Network (CHINET), the prevalence of clinical LRE was 0.92% (1.9% for *E. faecalis* and 0.2% for *E. faecium*) involving 44 Chinese hospitals in 2018 (Fupin et al., 2020), and increased to 1.9% (3.8% for *E. faecalis* and 0.4% for *E. faecium*) involving 73 hospitals in 2023 (Fupin et al., 2023). However, the LRE carriage in the community population in this study reached 6.01% (34/565, 2018–2019), indicating that the prevalence of LRE in the Chinese healthy population might be underestimated. OPEs exhibited high resistance rates to linezolid, florfenicol, doxycycline, and erythromycin, with corresponding ARGs like *fexA* (96.08%), *cat* (45.10%), *tet*(M) (95.10%), *erm*(A) (61.76%), and *erm*(B) (86.27%) also showing high detection rates. Similar results - as the higher resistance rates in the *optrA*-positive group - were displayed for multiple types of farm samples in Vietnam (Ha et al., 2023). Our results indicated that not only high resistance rates but also a larger number of ARGs were presented in OPEs, suggesting that OPEs poses a higher risk to public health. Our previous study revealed that higher daily consumption of pork and hospitalization within 3 months were associated with a higher risk of *optrA* carriage in these healthy population (Xiang et al., 2022). Despite the heterogeneous population structure observed in *optrA*-positive *E. faecalis* in the community, high homology was observed in *optrA*-positive *E. faecalis* isolates between from the community and other sources (clinics, pigs, chickens, pets and environment), suggesting that multiple sources, including animals and clinics, contribute to the human *optrA* carriage.

Unlike studies on the presence of *optrA* in diverse ecological niches (Schwarz et al., 2021), comprehensive studies on *optrA*-bearing plasmids are still lacking. Diverse types of *optrA*-carrying plasmids were identified in this study, with the majority found in *E. faecalis*. Mostly *optrA*-carrying plasmids in *E. faecalis* carried the *rep*9 replicon, which belongs to the *rep*A_N replicon family and is considered specific for *E. faecalis* (Mikalsen et al., 2015). The *rep*A_N replicon is the most prevalent plasmid family in Gram-positive bacteria and belongs to narrow host range plasmids (Weaver et al., 2009). The *rep*9 family plasmids contained multiple characterized sex-pheromone response plasmids such as pAD1. The sex-pheromone based conjugation systems were known as very efficient transfer vehicles and could promote the transfer of ARGs (Jensen et al., 2010). All these observations might explain the high detection rate of *rep*9 replicons in *optrA*-positive *E. faecalis* in present study. Moreover, pAD1(*rep*9 family) + DOp1-type *optrA*-carrying plasmids were found to be the most prevalent in the present study and were detected exclusively in *E. faecalis*, which was in agreement with the results of Nuesch-Inderbinen et al., who identified *optrA*-carrying plasmids in florfenicol-resistant *E. faecalis* isolated from beef cattle (Nuesch-Inderbinen et al., 2023). Moreover, the pAD1 + DOp1 *optrA*-carrying plasmids were highly similarly to those from various sources deposited in the NCBI database. Given that the role of pAD1 + DOp1-type plasmids in helping the spread of *optrA* has not been reported, further investigation into the cross-host transmission of this plasmid type is warranted. The *rep*US1-containing *optrA*-carrying plasmid were widely detected in other enterococcal species except *E. faecalis*, especially pTEF1 + pVEF1(*rep*US1 family)-type plasmid pEAM528p carrying two linezolid resistance genes *cfr* and *optrA*. The *rep*US1 family plasmids belong to broad host Inc18 plasmid, which has been detected in a wide range of Gram-positive bacteria and was proved to transmit resistance across species, played an important role in ARGs spreading (Jensen et al., 2010; Wardal et al., 2022). In this study, it was found that six *rep*US1-containing plasmids were identified novel *optrA*-carrying plasmids, indicating that *rep*US1-containing *optrA*-carrying plasmids in other enterococcal species except *E. faecalis* need more research. Notably, multiple types of *optrA*-carrying plasmids showed that ARGs co-located with *optrA* accounted for more than 50% of the total ARGs carried by the isolates (83.87% for pAD1 + DOp1-type plasmids), indicating that with the acquisition of an *optrA*-carrying plasmid, other ARGs are likely to be co-acquired. It suggested that the acquisition of *optrA*-carrying plasmids will make humans more vulnerable to antimicrobial resistance, especially MLS_B_ (*erm*(A) and *erm*(B)), tetracycline (*tet*(M) and *tet*(L)), aminoglycoside (*aph(3′)-III*), bacitracin (*bcrABDR*), trimethoprim (*dfrG*), florfenicol (*fexA* and *cat*) and other antimicrobial resistance.

In this study, *optrA* was surrounded by several MGEs, in which IS*1216E* appeared most frequently and was consistent with the previously reported structures including IS*1216E*-*fexA-optrA-erm*(A)-IS*1216E*, IS*1216E-optrA-erm*(A)-IS*1216E* and IS*1216E-fexA-optrA*-IS*1216E* (Schwarz et al., 2021). In addition, IS*Enfa5*, linked to *cfr* (Morroni et al., 2018), may be related to *optrA* as well, as the similar genetic structure was only identified in the study of Weiyi Shen et al. (Shen et al., 2024). In agreement to a previous study (Wu et al., 2019), Tn*554*-related genetic environments of *optrA* were predominantly located on chromosomes. Notably, *optrA* was often co-located with the bacitracin resistance gene cluster *bcrABDR* as a form of IS*Enfa1-bcrABDR*-IS*Enfa1* or IS*1216E*-*bcrABDR*-IS*1216E*, both of which were located either up or downstream of *optrA*, and the most commonly observed structure was IS*Enfa1*-IS*1216E-fexA-optrA-ISEnfa1-bcrABDR*-IS*Enfa1* in present study (Figure 4A, Type 10). This structure was also observed in the porcine enterococcal plasmid pE035 (Hao et al., 2019). The transfer of *bcrABDR* has previously been identified to be possibly related to IS*Enfa1* and IS*1216E*, especially IS*Enfa1* (Matos et al., 2009; Wang et al., 2015; Chen et al., 2016; Huang et al., 2019; Xuan et al., 2023). As IS*Enfa1* or IS*1216E* were interspersed between *optrA* and *bcrABDR* in various forms in our study, and considering the high homology of IS*1216E* and IS*Enfa1* (94% aa similar), it is reasonable to speculate that IS*1216E* and IS*Enfa1* can mediate the transfer of *optrA*, *bcrABDR* or both in various forms. Bacitracin was widely used as a growth promoter and prophylactic agent in livestock, but generally used to treat local infections caused by Gram-positive bacteria and for occasional oral administration in human clinics (Wang et al., 2015; Tran et al., 2015). The consumption of bacitracin, as a growth promoter, was 1,352 tons and accounted for 4.12% of the total antimicrobial use in animals in China, 2020 (Ministry of Agriculture, 2021). Previous studies have characterized the acquisition of *bcrABDR* encoding high-level bacitracin resistance in *E. faecium* and *E. faecalis* (Wang et al., 2015; Manson et al., 2004). Thus, the extensive use of this antimicrobial agent in animals might have played a role as a vital driver for the co-transfer and prevalence of *optrA* and *bcrABDR* in human enterococci. It also suggested that the *optrA* is likely to spread from livestock to the human gut through the food chain. Moreover, IS*1216E+bcrABDR*-related genetic contexts of *optrA* could be located on a variety of plasmids and detected not only in *E. faecalis* but also in *E. casseliflavus*, which again highlighted the need to pay attention to the risk of the spread of *optrA* in the food chain. Moreover, phenicol resistance gene *fexA* and MLS_B_ resistance gene *erm*(A) were also frequently identified IS*1216E*-related genetic contexts of *optrA*, which was similar to previous study (Ha et al., 2023; He et al., 2016). The widespread use of florfenicol and MLS_B_ in animals could also contribute to the spread of *optrA* in the food chain.

In summary, the high prevalence of multi-drug resistant OPEs in the human intestinal flora indicated that community populations serve as a significant reservoir of *optrA*. OPEs showed high resistance rates to linezolid, florfenicol, doxycycline and erythromycin and corresponding ARGs including *fexA*, *cat*, *tet*(M), *erm*(A) and *erm*(B) also showed high detection rates. Antimicrobial resistance and presence of ARGs were higher in OPEs than that in ONEs. Specifically, *E. faecalis* poses a high risk for the spread of *optrA* in human communities, with larger numbers of ARGs and harboring most types of *optrA*-carrying plasmids. The *rep9*-containing *optrA*-carrying plasmids were often and only detected in *E. faecalis*. Meanwhile, *rep*US1-containing *optrA*-carrying plasmid were widely detected in other enterococcal species. Most ARGs harbored by OPEs were identified on *optrA*-carrying plasmids, suggesting that the acquisition of *optrA*-carrying plasmids will make humans more vulnerable to antimicrobial resistance, thereby posing a greater threat to public health. Notably, the pAD1(*rep*9a_1_*rep*A) + DOp1-type *optrA*-carrying plasmids should receive more attention for the transfer of *optrA* given their high prevalence (24/66, 36.36%), high number of co-located ARGs with *optrA* (83.87% of total ARGs) and presence in multiple sources. The mobility of *optrA* was mainly associated with multiple MGEs, including IS*1216E*, IS*Enfa1*, IS*Enfa5* and Tn*6674*. Tn*6674* are primarily located on chromosome but IS*1216E* or IS*Enfa1* are mostly located on the plasmids. The *bcrABDR* gene cluster, conferring resistance to the animal growth promoter bacitracin, was firstly frequently identified surrounding *optrA* in present study. It underscores the pressing need to closely monitor and mitigate the risk of optrA dissemination in the food chain. Given that linezolid is a last-resort antimicrobial agent for treating serious infections caused by Gram-positive bacteria, community-acquired *optrA*-positive enterococci and *optrA*-carrying plasmids and other origins, especially the *optrA*-positive *E. faecalis* and pAD1 + DOp1-type plasmids, should be incorporated into public health monitoring programs.

## Data Availability

The genomic sequences obtained in this study have been deposited in NCBI Genbank database under the BioProject accession number, PRJNA1159101 (https://www.ncbi.nlm.nih.gov/bioproject/PRJNA1159101).
