# Supplementary material for: High prevalence and plasmidome diversity of optrA-positive enterococci in a Shenzhen community, China
Source: Front Microbiol. 2024 Dec 20;15:1505107. doi: 10.3389/fmicb.2024.1505107 (PMC11695379; doi:10.3389/fmicb.2024.1505107)
Supplement: Supplementary file 1 [file Table_1.docx]

Supplementary Table 1: Antimicrobial susceptibility of *optrA-*positive and -negative enterococci from fecal samples

in community population (μg/mL)

| Number | Species | Group | LNZ | FFC | CIP | ERY | VAN | TGC | DOX | A/C | AMP | DAP | FM |
| --- | --- | --- | --- | --- | --- | --- | --- | --- | --- | --- | --- | --- | --- |
| 44p | *E. faecalis* | *optrA+* | 2 | 32 | 8 | >128 | 1 | ≤0.25 | ≤0.5 | ≤0.5/0.25 | ≤0.5 | 2 | ≤8 |
| 62p | *E. faecalis* | *optrA+* | 8 | 64 | 1 | >128 | 1 | ≤0.25 | 16 | ≤0.5/0.25 | ≤0.5 | 4 | ≤8 |
| 68p | *E. faecalis* | *optrA+* | 8 | 64 | 0.5 | >128 | 1 | ≤0.25 | 16 | ≤0.5/0.25 | ≤0.5 | 2 | ≤8 |
| 96p | *E. faecalis* | *optrA+* | 4 | 32 | 0.5 | >128 | 1 | ≤0.25 | 16 | ≤0.5/0.25 | ≤0.5 | 2 | ≤8 |
| 113p | *E. faecalis* | *optrA+* | 4 | 64 | 0.5 | >128 | 1 | ≤0.25 | 16 | ≤0.5/0.25 | ≤0.5 | 2 | ≤8 |
| 121p | *E. faecalis* | *optrA+* | 4 | 64 | 1 | >128 | 4 | ≤0.25 | 2 | ≤0.5/0.25 | ≤0.5 | 2 | ≤8 |
| 144p | *E. faecalis* | *optrA+* | 4 | 64 | 0.5 | >128 | 1 | ≤0.25 | 16 | ≤0.5/0.25 | ≤0.5 | 1 | ≤8 |
| 145p | *E. faecalis* | *optrA+* | 8 | 64 | 0.5 | >128 | 1 | ≤0.25 | 32 | ≤0.5/0.25 | ≤0.5 | 2 | ≤8 |
| 151p | *E. faecalis* | *optrA+* | 1 | 64 | 4 | >128 | 1 | ≤0.25 | 2 | ≤0.5/0.25 | ≤0.5 | ≤0.5 | ≤8 |
| 203p | *E. faecalis* | *optrA+* | 8 | 64 | 1 | >128 | 2 | ≤0.25 | 32 | ≤0.5/0.25 | ≤0.5 | 2 | ≤8 |
| 207p | *E. faecalis* | *optrA+* | 4 | 64 | 1 | >128 | 2 | ≤0.25 | 16 | ≤0.5/0.25 | ≤0.5 | 2 | ≤8 |
| 215p | *E. faecalis* | *optrA+* | 4 | 64 | 32 | >128 | 1 | ≤0.25 | 16 | ≤0.5/0.25 | ≤0.5 | 4 | ≤8 |
| 219p | *E. faecalis* | *optrA+* | 4 | 64 | 0.5 | >128 | 1 | ≤0.25 | 16 | ≤0.5/0.25 | ≤0.5 | 2 | ≤8 |
| 222p | *E. faecalis* | *optrA+* | 4 | 64 | 4 | >128 | 1 | ≤0.25 | 16 | ≤0.5/0.25 | ≤0.5 | 1 | ≤8 |
| 227p | *E. faecalis* | *optrA+* | 4 | 64 | ≤0.25 | >128 | 2 | ≤0.25 | 16 | ≤0.5/0.25 | ≤0.5 | 1 | ≤8 |
| 252p | *E. faecalis* | *optrA+* | 4 | 64 | 0.5 | >128 | 1 | ≤0.25 | 16 | ≤0.5/0.25 | ≤0.5 | 1 | ≤8 |
| 263p | *E. faecalis* | *optrA+* | 8 | 64 | 1 | >128 | 2 | ≤0.25 | 16 | ≤0.5/0.25 | ≤0.5 | 2 | ≤8 |
| 267p | *E. faecalis* | *optrA+* | 1 | 64 | ≤0.25 | >128 | 4 | ≤0.25 | 16 | ≤0.5/0.25 | ≤0.5 | 2 | ≤8 |
| 268p | *E. faecalis* | *optrA+* | 8 | 64 | 2 | >128 | 2 | ≤0.25 | 16 | ≤0.5/0.25 | ≤0.5 | 1 | ≤8 |
| 329p | *E. faecalis* | *optrA+* | 8 | 64 | 0.5 | >128 | 1 | ≤0.25 | 16 | ≤0.5/0.25 | ≤0.5 | 2 | ≤8 |
| 345p | *E. faecalis* | *optrA+* | 16 | 64 | 1 | >128 | 2 | ≤0.25 | 16 | ≤0.5/0.25 | ≤0.5 | 2 | ≤8 |
| 355p | *E. faecalis* | *optrA+* | 4 | 64 | <0.25 | >128 | 2 | ≤0.25 | 16 | ≤0.5/0.25 | ≤0.5 | 2 | ≤8 |
| 357p | *E. faecalis* | *optrA+* | 8 | 64 | 1 | >128 | 2 | ≤0.25 | 8 | ≤0.5/0.25 | ≤0.5 | 1 | ≤8 |
| 378p | *E. faecalis* | *optrA+* | 8 | 64 | 0.5 | 8 | 1 | ≤0.25 | 16 | ≤0.5/0.25 | ≤0.5 | 4 | ≤8 |
| 387p | *E. faecalis* | *optrA+* | 4 | 64 | 2 | 8 | 1 | ≤0.25 | 16 | ≤0.5/0.25 | ≤0.5 | 4 | ≤8 |
| 419p | *E. faecalis* | *optrA+* | 4 | 64 | 8 | >128 | 1 | ≤0.25 | 8 | ≤0.5/0.25 | ≤0.5 | 2 | ≤8 |
| 433p | *E. faecalis* | *optrA+* | 8 | 64 | 1 | >128 | 1 | ≤0.25 | 16 | ≤0.5/0.25 | ≤0.5 | 2 | ≤8 |
| 435p | *E. faecalis* | *optrA+* | 8 | 32 | 1 | >128 | 2 | ≤0.25 | 32 | ≤0.5/0.25 | ≤0.5 | 2 | ≤8 |
| 445p | *E. faecalis* | *optrA+* | 8 | 64 | 1 | 16 | 1 | ≤0.25 | 32 | ≤0.5/0.25 | ≤0.5 | 4 | ≤8 |
| 446p | *E. faecalis* | *optrA+* | 4 | 64 | 1 | >128 | 1 | ≤0.25 | 16 | ≤0.5/0.25 | ≤0.5 | 2 | ≤8 |
| 473p | *E. faecalis* | *optrA+* | 8 | 32 | 16 | >128 | 1 | ≤0.25 | 16 | ≤0.5/0.25 | ≤0.5 | 2 | ≤8 |
| 478p | *E. faecalis* | *optrA+* | 2 | 32 | 1 | >128 | 1 | ≤0.25 | 16 | ≤0.5/0.25 | ≤0.5 | 2 | ≤8 |
| 497p | *E. faecalis* | *optrA+* | 4 | 64 | 1 | >128 | 1 | ≤0.25 | 16 | ≤0.5/0.25 | ≤0.5 | 2 | ≤8 |
| 502p | *E. faecalis* | *optrA+* | 4 | 64 | 4 | >128 | 2 | ≤0.25 | 16 | ≤0.5/0.25 | ≤0.5 | 2 | ≤8 |
| 520p | *E. faecalis* | *optrA+* | 4 | 64 | 0.5 | >128 | 1 | ≤0.25 | 16 | ≤0.5/0.25 | ≤0.5 | 2 | ≤8 |
| 536p | *E. faecalis* | *optrA+* | 8 | 64 | 1 | >128 | 2 | ≤0.25 | 16 | ≤0.5/0.25 | ≤0.5 | ≤0.5 | ≤8 |
| 544p | *E. faecalis* | *optrA+* | 8 | 64 | 1 | >128 | 2 | ≤0.25 | 16 | ≤0.5/0.25 | ≤0.5 | 2 | ≤8 |
| 575p | *E. faecalis* | *optrA+* | 8 | 64 | 1 | >128 | 1 | ≤0.25 | 32 | ≤0.5/0.25 | ≤0.5 | 2 | ≤8 |
| 576p | *E. faecalis* | *optrA+* | 2 | 32 | 4 | >128 | 1 | ≤0.25 | 8 | ≤0.5/0.25 | ≤0.5 | 2 | ≤8 |
| 606p | *E. faecalis* | *optrA+* | 4 | 32 | 0.5 | >128 | 1 | ≤0.25 | 16 | ≤0.5/0.25 | ≤0.5 | 2 | ≤8 |
| 608p | *E. faecalis* | *optrA+* | 4 | 64 | 4 | >128 | 1 | ≤0.25 | 16 | ≤0.5/0.25 | ≤0.5 | 4 | ≤8 |
| 628p | *E. faecalis* | *optrA+* | 4 | 32 | 0.5 | >128 | 1 | ≤0.25 | 16 | ≤0.5/0.25 | ≤0.5 | 1 | ≤8 |
| 655p | *E. faecalis* | *optrA+* | 4 | 64 | 1 | >128 | 4 | ≤0.25 | 16 | ≤0.5/0.25 | ≤0.5 | 2 | ≤8 |
| 663p | *E. faecalis* | *optrA+* | 8 | 64 | 1 | >128 | 2 | ≤0.25 | 16 | ≤0.5/0.25 | ≤0.5 | 2 | ≤8 |
| 671p | *E. faecalis* | *optrA+* | 8 | 64 | 16 | >128 | 1 | ≤0.25 | 8 | ≤0.5/0.25 | ≤0.5 | 2 | ≤8 |
| 697p | *E. faecalis* | *optrA+* | 4 | 64 | 1 | >128 | 1 | ≤0.25 | 8 | ≤0.5/0.25 | ≤0.5 | 1 | ≤8 |
| 698p | *E. faecalis* | *optrA+* | 8 | 64 | 16 | >128 | ≤0.5 | ≤0.25 | 8 | ≤0.5/0.25 | ≤0.5 | 1 | ≤8 |
| 709p | *E. faecalis* | *optrA+* | 8 | 64 | 16 | >128 | 1 | ≤0.25 | 16 | ≤0.5/0.25 | ≤0.5 | 2 | 32 |
| 712p | *E. faecalis* | *optrA+* | 8 | 64 | 0.5 | >128 | 1 | ≤0.25 | 16 | ≤0.5/0.25 | ≤0.5 | 1 | ≤8 |
| 713p | *E. faecalis* | *optrA+* | 4 | 64 | 0.5 | >128 | 1 | ≤0.25 | 16 | ≤0.5/0.25 | 1 | 2 | ≤8 |
| 715p | *E. faecalis* | *optrA+* | 8 | 64 | 32 | >128 | 1 | ≤0.25 | 8 | ≤0.5/0.25 | ≤0.5 | 2 | ≤8 |
| 723p | *E. faecalis* | *optrA+* | 8 | 64 | 0.5 | ≤0.5 | ≤0.5 | ≤0.25 | 16 | ≤0.5/0.25 | ≤0.5 | 1 | ≤8 |
| 730p | *E. faecalis* | *optrA+* | 8 | 64 | 32 | >128 | 1 | ≤0.25 | 32 | ≤0.5/0.25 | ≤0.5 | 4 | ≤8 |
| 738p | *E. faecalis* | *optrA+* | 4 | 64 | 0.5 | >128 | 2 | ≤0.25 | 16 | ≤0.5/0.25 | ≤0.5 | 2 | ≤8 |
| 739p | *E. faecalis* | *optrA+* | 4 | 32 | 0.5 | >128 | 1 | ≤0.25 | 8 | ≤0.5/0.25 | ≤0.5 | 2 | ≤8 |
| 740p | *E. faecalis* | *optrA+* | 4 | 64 | ≤0.25 | >128 | 2 | ≤0.25 | 16 | ≤0.5/0.25 | ≤0.5 | 2 | ≤8 |
| 746p | *E. faecalis* | *optrA+* | 2 | 32 | 0.5 | >128 | 2 | ≤0.25 | 8 | ≤0.5/0.25 | ≤0.5 | 2 | ≤8 |
| 752p | *E. faecalis* | *optrA+* | 4 | 64 | 0.5 | >128 | 2 | ≤0.25 | 16 | ≤0.5/0.25 | ≤0.5 | 2 | ≤8 |
| 760p | *E. faecalis* | *optrA+* | 4 | 64 | 0.5 | >128 | 1 | ≤0.25 | 16 | ≤0.5/0.25 | ≤0.5 | 2 | ≤8 |
| 784p | *E. faecalis* | *optrA+* | 4 | 64 | 0.5 | >128 | 2 | ≤0.25 | 16 | ≤0.5/0.25 | ≤0.5 | 1 | ≤8 |
| 798p | *E. faecalis* | *optrA+* | 2 | 16 | 16 | >128 | 2 | ≤0.25 | 16 | ≤0.5/0.25 | ≤0.5 | 4 | ≤8 |
| 838p | *E. faecalis* | *optrA+* | 4 | 64 | 0.5 | >128 | 1 | ≤0.25 | 16 | ≤0.5/0.25 | ≤0.5 | 2 | ≤8 |
| 869p | *E. faecalis* | *optrA+* | 4 | 64 | 0.5 | >128 | 1 | ≤0.25 | 16 | ≤0.5/0.25 | ≤0.5 | 4 | ≤8 |
| 871p | *E. faecalis* | *optrA+* | 8 | 64 | 32 | >128 | 1 | ≤0.25 | 16 | ≤0.5/0.25 | ≤0.5 | 2 | ≤8 |
| 876p | *E. faecalis* | *optrA+* | 8 | 64 | 0.5 | >128 | 1 | ≤0.25 | 8 | ≤0.5/0.25 | ≤0.5 | 1 | ≤8 |
| 886p | *E. faecalis* | *optrA+* | 4 | 32 | 16 | >128 | 1 | ≤0.25 | 8 | ≤0.5/0.25 | ≤0.5 | 1 | ≤8 |
| 887p | *E. faecalis* | *optrA+* | 4 | 64 | 0.5 | >128 | 1 | ≤0.25 | 16 | ≤0.5/0.25 | ≤0.5 | 2 | ≤8 |
| 889p | *E. faecalis* | *optrA+* | 4 | 64 | 0.5 | >128 | 2 | ≤0.25 | 16 | ≤0.5/0.25 | ≤0.5 | 1 | ≤8 |
| 899p | *E. faecalis* | *optrA+* | 8 | 64 | 0.5 | 2 | 1 | ≤0.25 | 16 | ≤0.5/0.25 | ≤0.5 | 2 | ≤8 |
| 915p | *E. faecalis* | *optrA+* | 8 | 64 | 16 | >128 | 2 | ≤0.25 | 16 | ≤0.5/0.25 | ≤0.5 | 1 | ≤8 |
| 942p | *E. faecalis* | *optrA+* | 4 | 64 | 16 | >128 | 1 | ≤0.25 | 16 | ≤0.5/0.25 | ≤0.5 | 1 | ≤8 |
| 958p | *E. faecalis* | *optrA+* | 8 | 64 | 0.5 | >128 | 1 | ≤0.25 | 16 | ≤0.5/0.25 | ≤0.5 | 2 | ≤8 |
| 973p | *E. faecalis* | *optrA+* | 4 | 64 | 0.5 | >128 | 1 | ≤0.25 | 16 | ≤0.5/0.25 | ≤0.5 | 2 | ≤8 |
| 987p | *E. faecalis* | *optrA+* | 4 | 64 | 1 | >128 | 2 | ≤0.25 | 16 | ≤0.5/0.25 | ≤0.5 | 2 | ≤8 |
| 999p | *E. faecalis* | *optrA+* | 8 | 32 | 0.5 | >128 | 1 | ≤0.25 | 16 | ≤0.5/0.25 | ≤0.5 | 2 | ≤8 |
| 339p | *E. avium* | *optrA+* | 2 | 64 | 8 | >128 | 1 | ≤0.25 | 2 | ≤0.5/0.25 | ≤0.5 | ≤0.5 | ≤8 |
| 422p | *E. avium* | *optrA+* | 2 | 64 | 2 | >128 | 1 | ≤0.25 | 16 | ≤0.5/0.25 | ≤0.5 | ≤0.5 | 32 |
| 524p | *E. avium* | *optrA+* | 2 | 64 | 2 | >128 | 1 | ≤0.25 | 16 | ≤0.5/0.25 | ≤0.5 | ≤0.5 | 32 |
| 528p | *E. avium* | *optrA+* | 2 | 32 | 16 | >128 | 1 | ≤0.25 | 8 | ≤0.5/0.25 | ≤0.5 | 1 | ≤8 |
| 934p | *E. avium* | *optrA+* | 2 | 32 | 2 | >128 | 1 | ≤0.25 | 16 | ≤0.5/0.25 | ≤0.5 | ≤0.5 | 32 |
| 959p | *E. avium* | *optrA+* | 2 | 64 | 1 | >128 | ≤0.5 | ≤0.25 | 16 | 8/4 | 8 | ≤0.5 | 16 |
| 83p | *E. casseliflavus* | *optrA+* | 4 | 128 | ≤0.25 | >128 | 4 | ≤0.25 | 2 | ≤0.5/0.25 | ≤0.5 | 2 | ≤8 |
| 166p | *E. casseliflavus* | *optrA+* | 4 | 64 | 0.5 | >128 | 1 | ≤0.25 | 16 | ≤0.5/0.25 | ≤0.5 | 2 | ≤8 |
| 298p | *E. casseliflavus* | *optrA+* | 4 | 64 | 32 | >128 | 1 | ≤0.25 | 8 | ≤0.5/0.25 | ≤0.5 | 1 | ≤8 |
| 531p | *E. casseliflavus* | *optrA+* | 8 | 64 | 4 | >128 | 1 | ≤0.25 | 4 | ≤0.5/0.25 | ≤0.5 | 2 | ≤8 |
| 862p | *E. casseliflavus* | *optrA+* | 2 | 128 | 4 | >128 | 4 | ≤0.25 | 16 | ≤0.5/0.25 | ≤0.5 | 1 | ≤8 |
| 957p | *E. casseliflavus* | *optrA+* | 4 | 128 | 0.5 | >128 | 1 | ≤0.25 | 4 | ≤0.5/0.25 | ≤0.5 | 2 | ≤8 |
| 279p | *E. faecium* | *optrA+* | 4 | 64 | 2 | >128 | 4 | ≤0.25 | 8 | ≤0.5/0.25 | 1 | 1 | ≤8 |
| 315p | *E. faecium* | *optrA+* | 1 | 64 | 1 | >128 | 1 | ≤0.25 | 8 | ≤0.5/0.25 | ≤0.5 | 1 | ≤8 |
| 423p | *E. faecium* | *optrA+* | 2 | 64 | 2 | >128 | 1 | ≤0.25 | 16 | ≤0.5/0.25 | ≤0.5 | ≤0.5 | 32 |
| 522p | *E. faecium* | *optrA+* | 4 | 32 | 16 | >128 | ≤0.5 | ≤0.25 | 8 | ≤0.5/0.25 | ≤0.5 | 2 | ≤8 |
| 589p | *E. faecium* | *optrA+* | 0.5 | 32 | 2 | >128 | <1 | ≤0.25 | 16 | ≤0.5/0.25 | ≤0.5 | 1 | ≤8 |
| 789p | *E. faecium* | *optrA+* | 2 | 32 | 1 | >128 | ≤0.5 | ≤0.25 | 16 | ≤0.5/0.25 | ≤0.5 | 1 | ≤8 |
| 966p | *E. faecium* | *optrA+* | 8 | >128 | 32 | >128 | ≤0.5 | ≤0.25 | 16 | ≤0.5/0.25 | 1 | ≤0.5 | 32 |
| 161p | *E. gallinarum* | *optrA+* | 2 | 64 | ≤0.25 | >128 | 4 | ≤0.25 | 4 | ≤0.5/0.25 | 1 | 1 | ≤8 |
| 574p | *E. gallinarum* | *optrA+* | 8 | 32 | 32 | >128 | 8 | ≤0.25 | 4 | ≤0.5/0.25 | ≤0.5 | 2 | ≤8 |
| 891p | *E. gallinarum* | *optrA+* | 2 | 64 | 4 | >128 | 4 | ≤0.25 | 8 | ≤0.5/0.25 | ≤0.5 | 1 | ≤8 |
| 933p | *E. gallinarum* | *optrA+* | 2 | 64 | 1 | >128 | 4 | ≤0.25 | 4 | ≤0.5/0.25 | ≤0.5 | 2 | ≤8 |
| 323p | *E. hirae* | *optrA+* | 4 | 64 | 2 | 4 | 1 | ≤0.25 | 64 | ≤0.5/0.25 | ≤0.5 | 1 | ≤8 |
| 726p | *E. hirae* | *optrA+* | 2 | 16 | 0.5 | 2 | ≤0.5 | ≤0.25 | 16 | ≤0.5/0.25 | ≤0.5 | 1 | 32 |
| 785p | *E. hirae* | *optrA+* | 1 | 16 | ≤0.25 | >128 | ≤0.5 | ≤0.25 | 4 | ≤0.5/0.25 | ≤0.5 | ≤0.25 | ≤8 |
| 943p | *E. hirae* | *optrA+* | 2 | 64 | 1 | >128 | ≤0.5 | ≤0.25 | 8 | ≤0.5/0.25 | 1 | 1 | ≤8 |
| 62n | *E. faecalis* | *optrA-* | 1 | 2 | 1 | ≤0.5 | 1 | <0.25 | ≤0.5 | ≤0.5/0.25 | ≤0.5 | 2 | ≤8 |
| 145n | *E. faecalis* | *optrA-* | 2 | 2 | 0.5 | 2 | 1 | <0.25 | ≤0.5 | ≤0.5/0.25 | ≤0.5 | 2 | ≤8 |
| 203n | *E. faecalis* | *optrA-* | 2 | 2 | 1 | 2 | 1 | <0.25 | 16 | ≤0.5/0.25 | ≤0.5 | 2 | ≤8 |
| 215n | *E. faecalis* | *optrA-* | 2 | 2 | 1 | 1 | 2 | <0.25 | ≤0.5 | ≤0.5/0.25 | ≤0.5 | 2 | ≤8 |
| 219n | *E. faecalis* | *optrA-* | 2 | 2 | 0.5 | 4 | 1 | <0.25 | 8 | ≤0.5/0.25 | ≤0.5 | 2 | ≤8 |
| 222n | *E. faecalis* | *optrA-* | 2 | 2 | ≤0.25 | 2 | 2 | <0.25 | 8 | ≤0.5/0.25 | ≤0.5 | 2 | ≤8 |
| 227n | *E. faecalis* | *optrA-* | 1 | 2 | 0.5 | 2 | 1 | <0.25 | 32 | ≤0.5/0.25 | ≤0.5 | 2 | ≤8 |
| 263n | *E. faecalis* | *optrA-* | 2 | 2 | 1 | 2 | 2 | <0.25 | 4 | ≤0.5/0.25 | ≤0.5 | 4 | ≤8 |
| 267n | *E. faecalis* | *optrA-* | 1 | 2 | 1 | 2 | ≤0.5 | <0.25 | ≤0.5 | ≤0.5/0.25 | ≤0.5 | 2 | ≤8 |
| 268n | *E. faecalis* | *optrA-* | 2 | 2 | 1 | ≤0.5 | 1 | <0.25 | ≤0.5 | ≤0.5/0.25 | ≤0.5 | 2 | ≤8 |
| 329n | *E. faecalis* | *optrA-* | 2 | 2 | 0.5 | 2 | 1 | <0.25 | ≤0.5 | ≤0.5/0.25 | ≤0.5 | 2 | ≤8 |
| 497n | *E. faecalis* | *optrA-* | 2 | 4 | 1 | >128 | 1 | <0.25 | 4 | ≤0.5/0.25 | ≤0.5 | 2 | ≤8 |
| 520n | *E. faecalis* | *optrA-* | 1 | 2 | 0.5 | ≤0.5 | 1 | <0.25 | 16 | ≤0.5/0.25 | ≤0.5 | 2 | ≤8 |
| 536n | *E. faecalis* | *optrA-* | ≤0.25 | ≤0.5 | ≤0.25 | 32 | 2 | <0.25 | 4 | ≤0.5/0.25 | ≤0.5 | 1 | ≤8 |
| 663n | *E. faecalis* | *optrA-* | 2 | 2 | 1 | 2 | 1 | <0.25 | 8 | ≤0.5/0.25 | ≤0.5 | 2 | ≤8 |
| 697n | *E. faecalis* | *optrA-* | 2 | 4 | 0.5 | >128 | 2 | <0.25 | 8 | ≤0.5/0.25 | ≤0.5 | 2 | ≤8 |
| 709n | *E. faecalis* | *optrA-* | 2 | 2 | 1 | 2 | 1 | <0.25 | ≤0.5 | ≤0.5/0.25 | ≤0.5 | 4 | ≤8 |
| 740n | *E. faecalis* | *optrA-* | 2 | 2 | 0.5 | 16 | 2 | <0.25 | 16 | ≤0.5/0.25 | ≤0.5 | 2 | ≤8 |
| 760n | *E. faecalis* | *optrA-* | 2 | 2 | 0.5 | ≤0.5 | 2 | <0.25 | ≤0.5 | ≤0.5/0.25 | ≤0.5 | 2 | ≤8 |
| 784n | *E. faecalis* | *optrA-* | 2 | 2 | 1 | >128 | 1 | <0.25 | ≤0.5 | ≤0.5/0.25 | ≤0.5 | 2 | ≤8 |
| 869n | *E. faecalis* | *optrA-* | ≤0.25 | ≤0.5 | 0.5 | ≤0.5 | 1 | <0.25 | ≤0.5 | ≤0.5/0.25 | ≤0.5 | 2 | ≤8 |
| 886n | *E. faecalis* | *optrA-* | 2 | 2 | 1 | 4 | 1 | <0.25 | 16 | ≤0.5/0.25 | ≤0.5 | 2 | ≤8 |
| 887n | *E. faecalis* | *optrA-* | 2 | 2 | 0.5 | ≤0.5 | 1 | <0.25 | ≤0.5 | ≤0.5/0.25 | ≤0.5 | 2 | ≤8 |
| 889n | *E. faecalis* | *optrA-* | 2 | 2 | 1 | 1 | 1 | <0.25 | ≤0.5 | ≤0.5/0.25 | ≤0.5 | 1 | ≤8 |
| 973n | *E. faecalis* | *optrA-* | 2 | 2 | 1 | 1 | 1 | <0.25 | ≤0.5 | ≤0.5/0.25 | ≤0.5 | 2 | ≤8 |
| 999n | *E. faecalis* | *optrA-* | 2 | 1 | 0.5 | 0.5 | 1 | <0.25 | 4 | ≤0.5/0.25 | ≤0.5 | 1 | ≤8 |

LNZ: Linezolid; FFC: Florfenicol; CIP: Ciprofloxacin; ERY: Erythromycin; VAN: Vancomycin; TGC: Tigecycline; DOX: Doxycycline; A/C: Amoxicillin-clavulanate; AMP: Ampicillin; DAP: Daptomycin; FM: Nitrofurantoin
