# Supplementary material for: High prevalence and plasmidome diversity of optrA-positive enterococci in a Shenzhen community, China
Source: Front Microbiol. 2024 Dec 20;15:1505107. doi: 10.3389/fmicb.2024.1505107 (PMC11695379; doi:10.3389/fmicb.2024.1505107)
Supplement: Supplementary file 2 [file Table_2.docx]

Supplementary Table 2: Antimicrobial susceptibility of *optrA*-positive and -negative enterococci from faecal samples in the community population

| Antimicrobial agents | *optrA*-_Match (n=26) | | | | *optrA+_*Match (n=26) | | | | *optrA*+_Total (n=102) | | | |
| --- | --- | --- | --- | --- | --- | --- | --- | --- | --- | --- | --- | --- |
|  | MIC range (µg/ml) | MIC_50_ (µg/ml) | MIC_90_ (µg/ml) | Resistance  rate | MIC range (µg/ml) | MIC_50_ (µg/ml) | MIC_90_ (µg/ml) | Resistance  rate | MIC range (µg/ml) | MIC_50_ (µg/ml) | MIC_90_ (µg/ml) | Resistance  rate |
| LNZ | [≤0.25 - 2] | 2 | 2 | 0 | [1 - 8] | 4 | 8 | 38.5% (10) | [0.5 - 16] | 4 | 8 | 33.3% (34) |
| FFC | [≤0.5 - 4] | 2 | 2 | 0 | [32 - 64] | 64 | 64 | 100% (26) | [>128 - 64] | 64 | 64 | 100.0% (102) |
| CIP | [≤0.25 - 1] | 1 | 1 | 0 | [≤0.25 - 32] | 0.5 | 4 | 15.4% (4) | [<0.25 - 32] | 1 | 16 | 27.5% (28) |
| ERY | [≤0.5 - >128] | 2 | >128 | 15.4% (4) | [>128] | >128 | >128 | 100% (26) | [≤0.5 - >128] | >128 | >128 | 97.0% (98) |
| VAN | [≤0.5 - 2] | 1 | 2 | 0 | [1 - 4] | 1 | 2 | 0 | [≤0.5 - 8] | 1 | 4 | 0 |
| TGC | [<0.25] | <0.25 | <0.25 | 0 | [<0.25] | <0.25 | <0.25 | 0 | [≤0.25] | ≤0.25 | ≤0.25 | 0 |
| DOX | [≤0.5 - 8] | ≤0.5 | 16 | 19.2% (5) | [8 - 32] | 16 | 16 | 88.5% (23) | [≤0.5 - 16] | 16 | 16 | 63.7% (65) |
| A/C | [≤0.5/0.25] | ≤0.5/0.25 | ≤0.5/0.25 | 0 | [≤0.5/0.25] | ≤0.5/0.25 | ≤0.5/0.25 | 0 | [≤0.5/0.25 - 8/4] | ≤0.5/0.25 | ≤0.5/0.25 | 0 |
| AMP | [≤0.5] | ≤0.5 | ≤0.5 | 0 | [≤0.5] | ≤0.5 | ≤0.5 | 0 | [≤0.5 - 8] | ≤0.5 | ≤0.5 | 0 |
| DAP | [1 - 4] | 2 | 2 | 0 | [<0.5 - 4] | 2 | 4 | 0 | [≤0.25 - 4] | 2 | 4 | 0 |
| FM | [≤8] | ≤8 | ≤8 | 0 | [≤8 - 32] | ≤8 | ≤8 | 0 | [≤8 - 32] | ≤8 | ≤8 | 0 |

LNZ: Linezolid; FFC: Florfenicol; CIP: Ciprofloxacin; ERY: Erythromycin; VAN: Vancomycin; TGC: Tigecycline; DOX: Doxycycline; A/C: Amoxicillin-clavulanate; AMP: Ampicillin; DAP: Daptomycin; FM: Nitrofurantoin
