# Supplementary material for: High prevalence and plasmidome diversity of optrA-positive enterococci in a Shenzhen community, China
Source: Front Microbiol. 2024 Dec 20;15:1505107. doi: 10.3389/fmicb.2024.1505107 (PMC11695379; doi:10.3389/fmicb.2024.1505107)
Supplement: Supplementary file 3 [file Table_3.docx]

Supplementary Table 3: Antimicrobial susceptibility of *optrA*-positive enterococci of different species from faecal samples in community population

|  | *E. faecalis* (n=75) | | | | *E. faecium* (n=7) | | | | Other enterococal species (n=20) | | | | Total (n=102) | | | |
| --- | --- | --- | --- | --- | --- | --- | --- | --- | --- | --- | --- | --- | --- | --- | --- | --- |
| Antimicrobial agents | MIC range (µg/ml) | MIC_50_ (µg/ml) | MIC_90_ (µg/ml) | Resistance  rate | MIC range (µg/ml) | MIC_50_ (µg/ml) | MIC_90_ (µg/ml) | Resistance  rate | MIC range (µg/ml) | MIC_50_ (µg/ml) | MIC_90_ (µg/ml) | Resistance  rate | MIC range (µg/ml) | MIC_50_ (µg/ml) | MIC_90_ (µg/ml) | Resistance  rate |
| LNZ | [1 - 16] | 4 | 8 | 41.3% (31) | [0.5 - 8] | 2 | 8 | 14.3% (1) | [1 - 8] | 2 | 7.6 | 10.0% (2) | [0.5 - 16] | 4 | 8 | 33.3% (34) |
| FFC | [16 - 64] | 64 | 64 | 100.0% (75) | [>128 - 64] | 48 | 64 | 100.0% (7) | [128 - 64] | 64 | 128 | 100.0% (20) | [>128 - 64] | 64 | 64 | 100.0% (102) |
| CIP | [<0.25 - 8] | 1 | 16 | 25.3% (19) | [1 - 32] | 2 | 32 | 28.6% (2) | [≤0.25 - 8] | 2 | 32 | 35.0% (7) | [≤0.25 - 8] | 1 | 16 | 27.5% (28) |
| ERY | [≤0.5 - >128] | 8 | 16 | 98.6% (73) | [>128] | >128 | >128 | 100.0% (7) | [2 ->128 ] | >128 | 4 | 90.0% (18) | [≤0.5 - >128] | >128 | 16 | 97.0% (98) |
| VAN | [≤0.5 - 4] | 1 | 2 | 0 | [≤0.5 - 4] | 1 | 4 | 0 | [≤0.5 - 8] | 1 | 1 | 0 | [≤0.5 - 8] | 1 | 4 | 0 |
| TGC | [≤0.25] | ≤0.25 | ≤0.25 | 0 | [≤0.25] | ≤0.25 | NA | 0 | [≤0.25] | ≤0.25 | ≤0.25 | 0 | [≤0.25] | ≤0.25 | ≤0.25 | 0 |
| DOX | [≤0.5 - 8] | 16 | 16 | 74.7% (56) | [16 - 8] | 16 | 16 | 28.6% (2) | [16 - 8] | 8 | 16 | 35.0% (7) | [≤0.5 - 8] | 16 | 16 | 63.7%(65) |
| A/C | [≤0.5/0.25] | ≤0.5/0.25 | ≤0.5/0.25 | 0 | [≤0.5/0.25] | ≤0.5/0.25 | ≤0.5/0.25 | 0 | [≤0.5/0.25 - 8/4] | ≤0.5/0.25 | ≤0.5/0.25 | 0 | [≤0.5/0.25 - 8/4] | ≤0.5/0.25 | ≤0.5/0.25 | 0 |
| AMP | [≤0.5 - 1] | 1 | 1 | 0 | [≤0.5 - 1] | 1 | 1 | 0 | [≤0.5 - 8] | 1 | 8 | 0 | [≤0.5 - 8] | 1 | 8 | 0 |
| DAP | [≤0.5 - 4] | 2 | 4 | 0 | [≤0.5 - 2] | 1 | 2 | 0 | [≤0.25 - 2] | 1 | 2 | 0 | [≤0.25 - 4] | 2 | 4 | 0 |
| FM | [≤8 - 32] | 32 | 32 | 0 | [≤8 - 32] | 32 | 32 | 0 | [≤8 - 32] | 32 | 32 | 0 | [≤8 - 32] | 32 | 32 | 0 |

LNZ: Linezolid; FFC: Florfenicol; CIP: Ciprofloxacin; ERY: Erythromycin; VAN: Vancomycin; TGC: Tigecycline; DOX: Doxycycline; A/C: Amoxicillin-clavulanate; AMP: Ampicillin; DAP: Daptomycin; FM: Nitrofurantoin
