# Supplementary material for: High prevalence and plasmidome diversity of optrA-positive enterococci in a Shenzhen community, China
Source: Front Microbiol. 2024 Dec 20;15:1505107. doi: 10.3389/fmicb.2024.1505107 (PMC11695379; doi:10.3389/fmicb.2024.1505107)
Supplement: Supplementary file 6 [file Table_6.docx]

Supplementary Table 6: MLST typing of enterococcal isolates in this study and detail profiles of *optrA*-carrying plasmids

| Number | Species | Group | MLST Typing | Plasmid Typing | Contig Length(bp) | Circular |
| --- | --- | --- | --- | --- | --- | --- |
| 44p | *E. faecalis* | *optrA+* | - | pAD1 | 55,282 | Yes |
| 62p | *E. faecalis* | *optrA+* | ST236 | pAD1+DOp1 | 68,206 | Yes |
| 68p | *E. faecalis* | *optrA+* | - | EF62pC+pSMA198 | 92,837 | Yes |
| 96p | *E. faecalis* | *optrA+* | ST631 | Chromosome | 2,714,812 | Yes |
| 113p | *E. faecalis* | *optrA+* | ST256 | Chromosome | 2,951,903 | Yes |
| 121p | *E. faecalis* | *optrA+* | ST632 | None | 12,141 | No |
| 144p | *E. faecalis* | *optrA+* | ST93 | pAD1+DOp1 | 68,893 | Yes |
| 145p | *E. faecalis* | *optrA+* | - | pAD1+DOp1 | 69,059 | Yes |
| 151p | *E. faecalis* | *optrA+* | ST16 | pTW9 | 35,923 | Yes |
| 203p | *E. faecalis* | *optrA+* | - | pAD1+DOp1 | 69,045 | Yes |
| 207p | *E. faecalis* | *optrA+* | - | EF62pC | 81,966 | Yes |
| 215p | *E. faecalis* | *optrA+* | - | Chromosome | 2,686,824 | Yes |
|  |  |  |  | pAD1+DOp1 | 58,553 | Yes |
| 219p | *E. faecalis* | *optrA+* | ST256 | pAD1+DOp1 | 68,849 | Yes |
| 222p | *E. faecalis* | *optrA+* | ST330 | pAD1+pSGG1 | 73,969 | Yes |
| 227p | *E. faecalis* | *optrA+* | ST93 | pAD1+DOp1 | 69,045 | Yes |
| 252p | *E. faecalis* | *optrA+* | ST376 | pAD1+DOp1 | 68,373 | Yes |
| 263p | *E. faecalis* | *optrA+* | ST116 | pAD1 | 38,588 | Yes |
| 267p | *E. faecalis* | *optrA+* | - | pAD1+DOp1 | 64,765 | Yes |
| 268p | *E. faecalis* | *optrA+* | ST93 | pAD1+DOp1 | 69,045 | Yes |
| 329p | *E. faecalis* | *optrA+* | ST16 | Chromosome | 2,998,141 | Yes |
| 345p | *E. faecalis* | *optrA+* | ST249 | pAD1 | 66,560 | Yes |
| 355p | *E. faecalis* | *optrA+* | ST34 | None | 74,496 | Yes |
| 357p | *E. faecalis* | *optrA+* | ST123 | None | 87,866 | Yes |
| 378p | *E. faecalis* | *optrA+* | ST633 | Chromosome | 2,691,228 | Yes |
| 387p | *E. faecalis* | *optrA+* | ST633 | Chromosome | 2,691,228 | Yes |
| 419p | *E. faecalis* | *optrA+* | ST16 | Chromosome | 2,923,609 | Yes |
| 433p | *E. faecalis* | *optrA+* | - | pAD1+DOp1 | 72,402 | Yes |
| 435p | *E. faecalis* | *optrA+* | ST409 | pAD1+DOp1 | 69,045 | Yes |
| 445p | *E. faecalis* | *optrA+* | ST376 | pAD1+DOp1 | 55,471 | Yes |
| 446p | *E. faecalis* | *optrA+* | ST632 | pTEF2 | 25,917 | Yes |
| 473p | *E. faecalis* | *optrA+* | ST16 | Chromosome | 3,021,424 | No |
| 478p | *E. faecalis* | *optrA+* | ST674 | None | 8,210 | No |
| 497p | *E. faecalis* | *optrA+* | - | pAD1+DOp1 | 69,862 | Yes |
| 502p | *E. faecalis* | *optrA+* | - | pTEF2 | 26,319 | Yes |
| 520p | *E. faecalis* | *optrA+* | ST59 | Chromosome | 2,761,867 | Yes |
| 536p | *E. faecalis* | *optrA+* | ST376 | pAD1+DOp1 | 69,022 | Yes |
| 544p | *E. faecalis* | *optrA+* | - | pAD1+DOp1 | 69,862 | Yes |
| 575p | *E. faecalis* | *optrA+* | ST538 | pSMA198+pAD1+DOp1 | 81,908 | Yes |
| 576p | *E. faecalis* | *optrA+* | - | Chromosome | 2,859,645 | Yes |
| 606p | *E. faecalis* | *optrA+* | ST376 | Chromosome | 2,848,804 | Yes |
| 608p | *E. faecalis* | *optrA+* | - | None | 72,284 | Yes |
| 628p | *E. faecalis* | *optrA+* | - | Chromosome | 3,106,482 | Yes |
| 655p | *E. faecalis* | *optrA+* | - | pAD1+DOp1 | 40,637 | No |
| 663p | *E. faecalis* | *optrA+* | - | pAD1+DOp1 | 69,045 | Yes |
| 671p | *E. faecalis* | *optrA+* | ST16 | Chromosome | 3,098,585 | Yes |
|  |  |  |  | EFD32pB | 52,932 | Yes |
| 697p | *E. faecalis* | *optrA+* | ST16 | None | 10,117 | No |
| 698p | *E. faecalis* | *optrA+* | ST330 | pAD1+DOp1 | 69,699 | Yes |
| 709p | *E. faecalis* | *optrA+* | ST256 | Chromosome | 2,827,243 | Yes |
| 712p | *E. faecalis* | *optrA+* | ST376 | pAD1+DOp1 | 68,849 | Yes |
| 713p | *E. faecalis* | *optrA+* | - | Chromosome | 2,976,562 | Yes |
| 715p | *E. faecalis* | *optrA+* | - | Chromosome | 2,453,129 | No |
|  |  |  |  | None | 8,181 | No |
| 723p | *E. faecalis* | *optrA+* | - | None | 6,627 | No |
| 730p | *E. faecalis* | *optrA+* | - | pTW9 | 71,574 | Yes |
| 738p | *E. faecalis* | *optrA+* | ST506 | pAD1+DOp1 | 67,821 | Yes |
| 739p | *E. faecalis* | *optrA+* | ST520 | pTEF2 | 70,868 | Yes |
| 740p | *E. faecalis* | *optrA+* | ST93 | EF62pC+DOp1 | 42,644 | No |
| 746p | *E. faecalis* | *optrA+* | ST618 | Chromosome | 2,834,798 | Yes |
| 752p | *E. faecalis* | *optrA+* | - | pAD1+DOp1 | 60,647 | Yes |
| 760p | *E. faecalis* | *optrA+* | - | pAD1+DOp1 | 87,783 | Yes |
| 784p | *E. faecalis* | *optrA+* | ST368 | None | 59,371 | Yes |
| 798p | *E. faecalis* | *optrA+* | ST403 | Chromosome | 2,933,471 | Yes |
| 838p | *E. faecalis* | *optrA+* | ST202 | None | 78,564 | Yes |
| 869p | *E. faecalis* | *optrA+* | ST593 | pAD1 | 72,033 | Yes |
| 871p | *E. faecalis* | *optrA+* | ST585 | pAD1+DOp1 | 83,234 | Yes |
| 876p | *E. faecalis* | *optrA+* | ST16 | None | 79,097 | Yes |
| 886p | *E. faecalis* | *optrA+* | ST632 | pTEF2 | 37,492 | Yes |
| 887p | *E. faecalis* | *optrA+* | - | Chromosome | 2,736,478 | Yes |
| 889p | *E. faecalis* | *optrA+* | ST618 | Chromosome | 2,841,644 | Yes |
| 899p | *E. faecalis* | *optrA+* | ST719 | pAD1 | 33,276 | Yes |
| 915p | *E. faecalis* | *optrA+* | ST59 | EF62pC+DOp1 | 67,128 | Yes |
| 942p | *E. faecalis* | *optrA+* | ST16 | pTW9 | 35,985 | Yes |
| 958p | *E. faecalis* | *optrA+* | ST16 | EF62pC+DOp1 | 56,271 | Yes |
| 973p | *E. faecalis* | *optrA+* | ST631 | Chromosome | 2,719,431 | Yes |
| 987p | *E. faecalis* | *optrA+* | ST202 | Chromosome | 2,832,613 | Yes |
| 999p | *E. faecalis* | *optrA+* | ST16 | EF62pC+DOp1 | 66,103 | Yes |
| 279p | *E. faecium* | *optrA+* | - | Chromosome | 2,505,290 | Yes |
| 315p | *E. faecium* | *optrA+* | ST66 | Chromosome | 2,514,291 | Yes |
| 423p | *E. faecium* | *optrA+* | ST55 | Chromosome | 2,642,468 | Yes |
| 522p | *E. faecium* | *optrA+* | - | Chromosome | 2,648,180 | Yes |
| 589p | *E. faecium* | *optrA+* | ST944 | Chromosome | 2,531,666 | Yes |
| 789p | *E. faecium* | *optrA+* | - | Chromosome | 2,489,497 | Yes |
| 966p | *E. faecium* | *optrA+* | ST194 | pVEF1 | 56,717 | Yes |
| 339p | *E. avium* | *optrA+* | - | Chromosome | 3,249,382 | No |
| 422p | *E. avium* | *optrA+* | - | Chromosome | 3,234,633 | Yes |
| 524p | *E. avium* | *optrA+* | - | Chromosome | 3,306,439 | Yes |
| 528p | *E. avium* | *optrA+* | - | pTEF1+pVEF1 | 64,541 | Yes |
| 934p | *E. avium* | *optrA+* | - | Chromosome | 3,195,724 | Yes |
| 959p | *E. avium* | *optrA+* | - | pVEF1 | 51,440 | Yes |
| 83p | *E. casseliflavus* | *optrA+* | - | pVEF3 | 62,313 | Yes |
| 166p | *E. casseliflavus* | *optrA+* | - | pVEF3 | 30,306 | Yes |
| 298p | *E. casseliflavus* | *optrA+* | - | None | 24,508 | No |
| 531p | *E. casseliflavus* | *optrA+* | - | None | 60,204 | Yes |
| 862p | *E. casseliflavus* | *optrA+* | - | pQY182 | 53,767 | Yes |
| 957p | *E. casseliflavus* | *optrA+* | - | pVEF1 | 56,300 | Yes |
| 161p | *E. gallinarum* | *optrA+* | - | Chromosome | 3,219,065 | No |
| 574p | *E. gallinarum* | *optrA+* | - | Chromosome | 3,284,599 | Yes |
| 891p | *E. gallinarum* | *optrA+* | - | Chromosome | 3,201,370 | Yes |
|  |  |  |  | DOp2 | 59,820 | Yes |
| 933p | *E. gallinarum* | *optrA+* | - | Chromosome | 3,264,692 | Yes |
| 323p | *E. hirae* | *optrA+* | - | Chromosome | 2,671,783 | Yes |
| 726p | *E. hirae* | *optrA+* | - | Chromosome | 2,676,653 | Yes |
| 785p | *E. hirae* | *optrA+* | - | Chromosome | 2,690,537 | Yes |
| 943p | *E. hirae* | *optrA+* | - | Chromosome | 2,680,711 | Yes |
| 62n | *E. faecalis* | *optrA-* | ST258 | - | - | - |
| 145n | *E. faecalis* | *optrA-* | ST116 | - | - | - |
| 203n | *E. faecalis* | *optrA-* | - | - | - | - |
| 215n | *E. faecalis* | *optrA-* | - | - | - | - |
| 219n | *E. faecalis* | *optrA-* | ST65 | - | - | - |
| 222n | *E. faecalis* | *optrA-* | - | - | - | - |
| 227n | *E. faecalis* | *optrA-* | ST918 | - | - | - |
| 263n | *E. faecalis* | *optrA-* | - | - | - | - |
| 267n | *E. faecalis* | *optrA-* | ST506 | - | - | - |
| 268n | *E. faecalis* | *optrA-* | ST998 | - | - | - |
| 329n | *E. faecalis* | *optrA-* | ST273 | - | - | - |
| 497n | *E. faecalis* | *optrA-* | ST6 | - | - | - |
| 520n | *E. faecalis* | *optrA-* | ST998 | - | - | - |
| 536n | *E. faecalis* | *optrA-* | ST47 | - | - | - |
| 663n | *E. faecalis* | *optrA-* | ST84 | - | - | - |
| 697n | *E. faecalis* | *optrA-* | ST4 | - | - | - |
| 709n | *E. faecalis* | *optrA-* | - | - | - | - |
| 740n | *E. faecalis* | *optrA-* | ST116 | - | - | - |
| 760n | *E. faecalis* | *optrA-* | ST21 | - | - | - |
| 784n | *E. faecalis* | *optrA-* | ST100 | - | - | - |
| 869n | *E. faecalis* | *optrA-* | ST409 | - | - | - |
| 886n | *E. faecalis* | *optrA-* | ST207 | - | - | - |
| 887n | *E. faecalis* | *optrA-* | ST593 | - | - | - |
| 889n | *E. faecalis* | *optrA-* | - | - | - | - |
| 973n | *E. faecalis* | *optrA-* | ST860 | - | - | - |
| 999n | *E. faecalis* | *optrA-* | - | - | - | - |
