# Supplementary material for: High prevalence and plasmidome diversity of optrA-positive enterococci in a Shenzhen community, China
Source: Front Microbiol. 2024 Dec 20;15:1505107. doi: 10.3389/fmicb.2024.1505107 (PMC11695379; doi:10.3389/fmicb.2024.1505107)
Supplement: Supplementary file 7 [file Data_Sheet_1.docx]

**List of Supplementary materials:**

**Supplementary Figure 1:** Comparison of VFGs number and VFGs profiles of *optrA*-positive and -negative enterococci (A,C), and of different enterococcal species (B,D).

a and b represent significant differences between the two groups; only asterisks indicated that the group showed significant differences with other groups；

“*” :*p* < 0.05; “**”:*p* < 0.01; “***”:*p* < 0.001.

**Supplementary Figure 2:** Plasmid maps of novel *optrA*-carrying plasmids with different types in *E. faecalis*. (A) plasmid pEF575p, pSMA198+pAD1+DOp1-type; (B) plasmid pEF68p, EF62pC+pSMA198-type; (C) plasmid pEF222p, pAD1+pSGG1-type; (D) plasmid pEF730p, pTW9-type; (E) plasmid pEF207p, EF62pC-type; (F) plasmid pEF71p, EFD32pB-type.

**Supplementary Figure 3:** Plasmid maps of novel *optrA*-carrying plasmids with different types in other enterococal species. (A) plasmid pEAM959p and pECS957p, pVEF1-type; (B) plasmid pECS166p and pECS83p, pVEF3-type; (C) plasmid pEAM528p, pTEF1+pVEF3-type; (D) plasmid pEGM891p, DOp2-type.

**Supplementary Figure 4:** Plasmid maps of novel *optrA*-carrying plasmids with no clear replicons identified in *E. faecalis* (A-C) and in *E. casseliflavus* (D).

**Supplementary Table 1:** Antimicrobial susceptibility of *optrA*-positive and -negative enterococci from fecal samples in community population (μg/mL).

**Supplementary Table 2:** Antimicrobial susceptibility of *optrA*-positive and -negative enterococci from fecal samples in the community population.

**Supplementary Table 3:** Antimicrobial susceptibility of *optrA*-positive enterococci of different species from fecal samples in community population.

**Supplementary Table 4:** ARGs profiles of *optrA*-positive and -negative enterococci isolated from fecal samples of the community population.

“1” indicated that the ARGs exists in the genome of isolates, and “0” indicated that ARGs doesn’t exist.

**Supplementary Table 5:** VFGs profiles of *optrA*-positive and -negative enterococci isolated from fecal samples of the community population.

“1” indicated that the VFGs exists in the genome of isolates, and “0” indicated that ARGs doesn’t exist.

**Supplementary Table 6:** MLST typing of enterococcal isolates in this study and detail profiles of *optrA*-carrying plasmids
