# Supplementary figures and images for: High prevalence and plasmidome diversity of optrA-positive enterococci in a Shenzhen community, China
Source: Front Microbiol. 2024 Dec 20;15:1505107. doi: 10.3389/fmicb.2024.1505107 (PMC11695379; doi:10.3389/fmicb.2024.1505107)

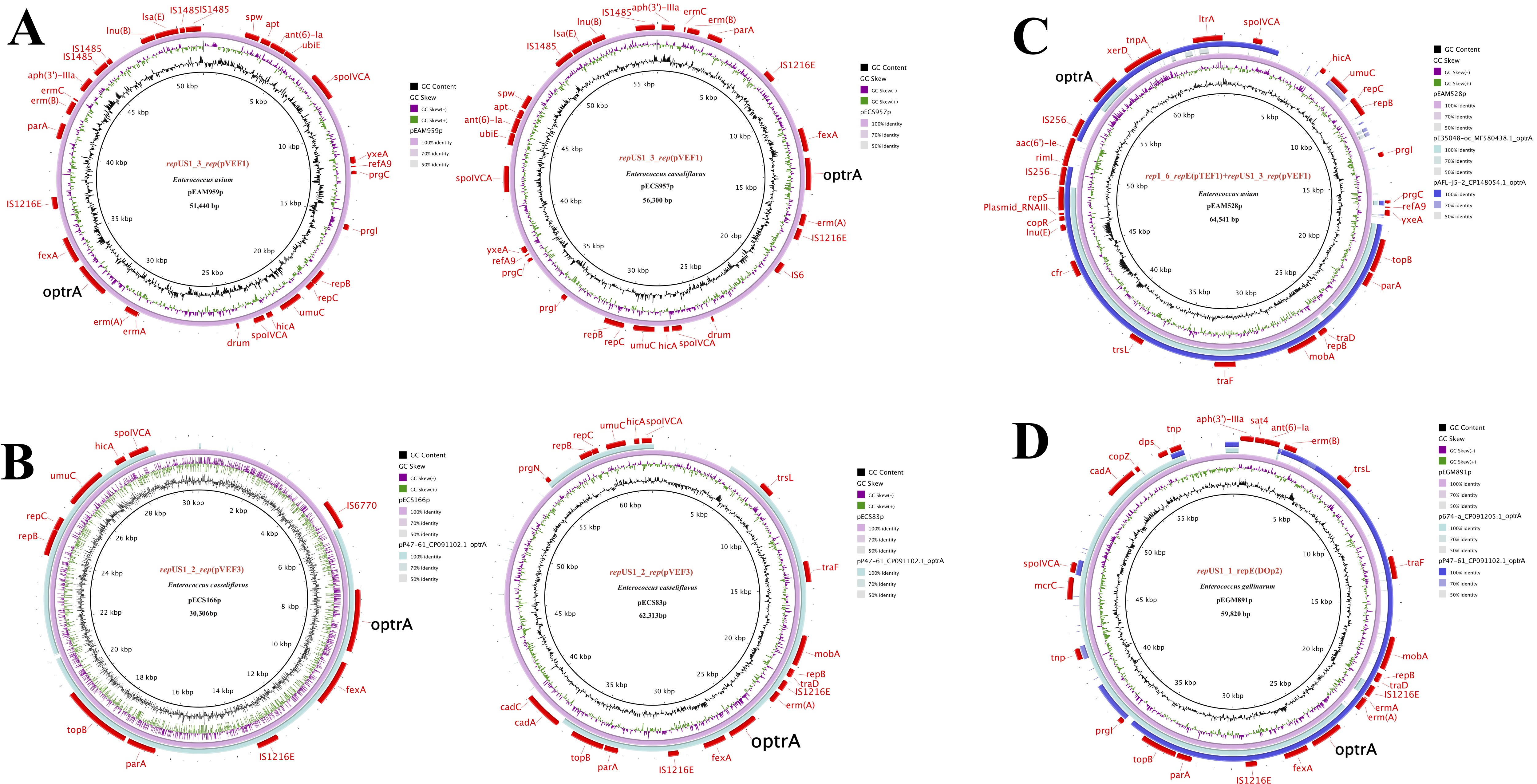

Supplement: Supplementary file 9 [file Image_3.jpeg]

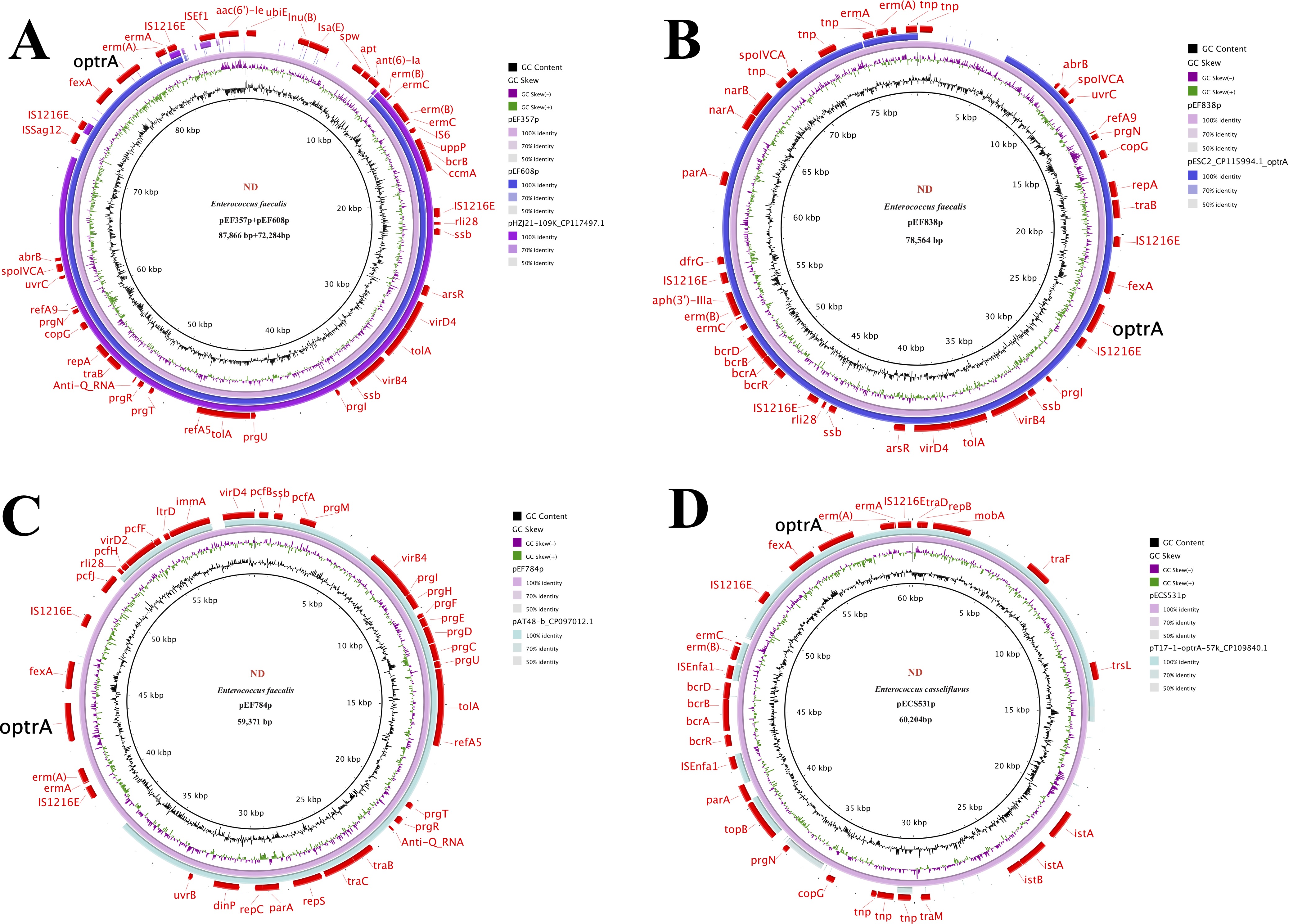

Supplement: Supplementary file 10 [file Image_4.jpeg]
